# PKI-587 enhances radiosensitization of hepatocellular carcinoma by inhibiting the PI3K/AKT/mTOR pathways and DNA damage repair

**DOI:** 10.1371/journal.pone.0258817

**Published:** 2021-10-19

**Authors:** Yinghai Xie, Changwei Liu, Yinci Zhang, Amin Li, Chong Sun, Rui Li, Yingru Xing, Minghong Shi, Qi Wang

**Affiliations:** 1 Huainan First People’s Hospital and First Affiliated Hospital of Medical School, Anhui University of Science and Technology, Huainan, China; 2 Medical School, Anhui University of Science and Technology, Huainan, China; 3 Institute of Environment-friendly Materials and Occupational Health of Anhui University of Science and Technology (Wuhu), Wuhu, China; 4 Affiliated Cancer Hospital, Anhui University of Science and Technology, Huainan, China; Chung Shan Medical University, TAIWAN

## Abstract

Radiation is an important therapeutic strategy for hepatocellular (HCC). In this study, we evaluated the role of the dual PI3K/mTOR inhibitor, PKI-587, on radiosensitization of HCC and its possible mechanism. MTT, colony formation, flow cytometry, and immunofluorescence were used to analyze the proliferation, cell cycle, formation of residual γ-H2AX foci, and apoptosis of HCC cells. A SK-Hep1 xenograft HCC model was used to assess the effects of PKI-587 in combination with ionizing radiation in vivo. The activation levels of PI3K/AKT/mTOR and DNA damage repair pathways and their downstream effector molecules were detected with Western blot. It was found that PKI-587 sensitized HCC cells to radiation by increasing DNA damage, enhancing G0/G1 cell-cycle arrest, and inducing apoptosis. In vivo, the combination of radiation with PKI-587 significantly inhibited tumor growth. These findings suggest the usefulness of PKI-587 on radiosensitization of HCC cells by inhibiting the PI3K/AKT/mTOR and DNA damage repair pathways. The combination of ionizing radiation and PKI-587 may be a strategy to improve the efficacy of treating HCC.

## Introduction

Hepatocellular carcinoma (HCC), with its high incidence and mortality, seriously threatens human health [1, 2]. One of the main treatments for HCC is surgical resection; another is local treatment, such as radiotherapy [3–5]. The toxicity and side effects of systemic treatment have led to an increased role of local treatment for HCC [6–8]. Radiotherapy, as a non-invasive treatment, plays a pivotal role in treatment of advanced HCC, which causes tumor-cell death by inducing apoptosis, mitotic death, autophagy, necrosis, and aging [9–12]. With the development of imaging technology, the precise dose and controllable range of radiation have a reasonable killing effect on tumor targets while minimizing the impact on surrounding normal tissues [13–15]. However, after being exposed to radiation, tumor cells can counteract the effects of radiation, thus decreasing the sensitivity of tumors to radiotherapy. Although several radiosensitizers have been reported [16–18], their therapeutic effect remains unsatisfactory, and there is an urgent need for more effective radiosensitizers.

Ionizing radiation (IR) can stimulate the abnormal activation of multiple signaling pathways that regulate tumor progression. Abnormal activation of the PI3K/AKT/mTOR signaling pathway is closely related to radiotherapy resistance by promoting proliferation, survival, metabolism, invasion, and metastasis by inhibiting autophagy and apoptosis in malignant tumors [19–23]. In addition, IR can trigger a series of DNA damage responses in tumor cells, such as cell-cycle arrest and activation of DNA damage repair pathways [24, 25]. The two main DNA double-strand break repair mechanisms are non-homologous end joining (NHEJ) and homologous recombination [26]. Crosstalk between the PI3K/AKT/mTOR and DNA damage repair pathways, mainly by interaction with damage response and repair (DDR) sensors/transducers, DDR mediators, and DDR effector molecules, has been reported [27]. Simultaneous inhibition of PI3K/AKT/mTOR and DNA damage repair pathways can enhance the chemotherapy sensitivity of HCC to platinum drugs (such as oxaliplatin), through a mechanism like that of radiation [28]. Whether simultaneous inhibition of the PI3K/AKT/mTOR and DNA damage repair pathways can enhance the radiotherapy sensitivity of HCC is unknown.

Drugs targeting the PI3K/AKT/mTOR signaling pathway have had good anti-proliferative, pro-apoptotic, and synergistic effects in tumor radiotherapy and chemotherapy [29–32]. PKI-587, an inhibitor targeting both PI3K and mTOR, can reduce the level of p-AKT and inhibit the proliferation and increase the radiosensitivity of tumor cells in vivo and in vitro [33–35]. In this study, we explored the effect of PKI-587 on the radiosensitivity and regulatory mechanisms in the HCC cell line SK-Hep1 in vivo and in vitro. We found that PKI-587, combined with radiation, enhanced the anti-tumor and radiosensitizing effects by inhibiting the PI3K/AKT/mTOR and DNA damage repair pathways. The results suggest a role for PKI-587 as a sensitizer in radiotherapy of HCC.

## Materials and methods

### Cell culture

Human HCC cell line SK-Hep1 was purchased from Blinded per Author Guidelines and cultured in RPMI 1640 medium and maintained in a 5% CO_2_ incubator at 37°C.

### Radiation and reagents

Radiation treatment was performed with a 6 MV-X ray source (Varian Medical Systems, California, USA), and the cells were irradiated at a dose rate of 0.2 Gy/min. PKI-587 (MeDeCm Express, USA) was dissolved in dimethyl sulfoxide at 2 mM and stored at -20°C. Antibodies to Ki67, PI3Kp110α, PI3Kp110γ, Akt, phospho-Akt (Ser473), mTOR, phospho-mTOR (Ser2448), Bad, phospho-Bad, Caspase-3, Cleaved Caspase-3, Caspase-9, Cleaved Caspase-9, PARP, and Cleaved RARP were purchased from Cell Signaling Technology, USA. Antibodies to phospho-Rb, Rb, Cyclin D1, phospho-DNAPKcs (Ser2056), DNAPKcs, phospho-ATM (Ser1981), ATM, phospho-ATR (Ser428), and ATR were acquired from Abcam, USA. Antibodies to p70S6K, phospho-S6K1 (T421 + S424), eIF4EBP1 and phospho-eIF4EBP1 were purchased from Boster Biological Technology, China. γ-H2AX was obtained from Biolegend Biological Technology, USA. β-actin was obtained from Biosharp Life Science, China. SP-9000 was obtained from Zhongshan Gold Bridge.

### Western blot analysis

Total protein was extracted after treating the cells in groups for 24 h, and protein concentration was determined with a BCA-200 Protein Assay Kit (Pierce, Rockford, IL, USA). Target proteins were separated by SDS-PAGE and electrotransferred onto polyvinylidene difluoride membranes (Millipore, USA). The membranes were blocked for 60 min with 5% skim milk in TBST (pH 8.3) at room temperature and incubated with primary antibody at 4°C for 12 h. The membranes were washed in TBST and incubated with secondary antibody (1:4000) for 60 min at room temperature followed by exposure to electrochemiluminescence.

### MTT assay

The cells were inoculated into 96-well plates at a final density of 5 × 10^3^ cells/well. After drug treatment of each group, the cell cultures were incubated with MTT solution (5 mg/mL) for 4 h at 37°C. The supernatant of each well was replaced by 150 μL dimethyl sulfoxide. A microplate reader (ELx800, Bio-Tek, Winooski, VT, USA) was used to detect the absorbance at 492 nm, and cell viability was calculated with Graphpad Prism Version 5.0 software according to absorbance.

### Clonogenic survival assay

Clonogenic survival assays were performed as described [36]. Cells were treated with PKI-587 (0.1 μM) for 6 h, then exposed to IR (0, 2, 4, 6, and 8 Gy) and washed for 24 h.

### Colony formation assay

SK-Hep1 cells were seeded into a six-well plate at a final density of 1000 cells/well. Cells were treated according to these conditions: Control, untreated; IR, 2 Gy 6 MV-X ray alone; PKI-587, 0.1μM PKI-587 alone; IR+PKI-587, combination IR + PKI-587. The cells were cultured until macroscopic clusters appeared in the plate. The cells were washed gently with PBS twice and fixed with 5% paraformaldehyde for 15 min, then stained with crystal violet for 20 min. Cell colonies were counted, and the number cultured with drugs was compared with the number cultured with untreated control.

### γ-H2AX assay

SK-Hep1 cells were seeded in 12-well plates with slides at a final density of 5000 cells per well. When the degree of cell fusion reached about 80%, the cells were treated with 2 Gy IR or 2 Gy IR+0.1 μM PKI-587 for 0, 12, 24, or 48 h. After rinsing with PBS for 3 min per time, the cells were fixed with 4% paraformaldehyde for 15 min. The slides were incubated with 0.5% Triton-100 for 30 min and blocked with 5% bovine serum albumin for 1 h. The γ-H2AX primary antibody (dilution, 1:100) was incubated at 37°C for 60 min. After washing with PBS 3 times, Cy3 fluorescent secondary antibody (1:1000) was added and incubated at 37°C for 60 min. The cells were counterstained with DAPI for 10 min. A fluorescence microscope was used to observe and photograph the cells.

### Cell cycle analysis

The cells were collected after treating as follows: Control, untreated; IR, 2Gy 6MV-Xray alone; PKI-587, 0.1 μM PKI-587 alone; IR+PKI-587, combination IR + PKI-587. The cells were dissociated and fixed with cold 75% ethanol at -20°C overnight. The ethanol was discarded after centrifuging, and the cells were washed with PBS. One hundred microliters of RNAse (100 μg/ml) and 400 μl propidium iodide (50 μg/ml) were added at room temperature in the dark for 30 min. The precipitate was collected, and 500 μL PBS was added to resuspend the cells for cell-cycle analysis by flow cytometry (BD FACSCalibur, USA).

### Annexin V-FITC/PI/DAPI staining

The cells were inoculated into a six-well plate containing cell slides at a final density of 1 × 10^4^ cells/well. After various treatments (Control, untreated; IR, 2 Gy 6 MV-Xray alone; PKI-587, 0.1uM PKI-587 alone; IR+PKI-587, combination IR + PKI-587), the slides were rinsed with PBS and stained with 200 μL PBS containing 10 μL Annexin V FITC, 3 μL propidium iodide, and 4 μL DAPI for 15 min in the dark. Fluorescence microscopy was used to detect the apoptotic rate.

### Measurement of mitochondrial membrane potential (JC-1)

The cells were inoculated on a 24-well plate at a final density of 5 × 10^5^ cells/mL. After various treatments (control, untreated; IR, 2 Gy 6MV-X ray alone; PKI-587, 0.1 μM PKI-587 alone; IR+PKI-587, combination IR + PKI-587), the cells were collected and incubated with 0.5 mL of 8 μg/mL JC-1 solution for 30 min. The cells were washed with PBS twice and re-suspended with 500 μL buffer solution for analysis by flow cytometry (BD FACSCalibur, USA).

### Acridine orange/ethidium bromide staining

The cells were inoculated on a 24-well plate with a density of 2 × 10^5^ cells/mL. After various treatments (control, untreated; IR, 2 Gy 6MV-Xray alone; PKI-587, 0.1 μM PKI-587 alone; IR+PKI-587, combination IR + PKI-587), the cells were washed with PBS twice and stained with 500 μL PBS containing 10 μL of the prepared working solution (acridine orange/ethidium bromide solution 1:1) for 5 min at 37°C. The stained cells were imaged under a fluorescence microscope.

### Xenograft studies

All the animal experiments were carried out in strict accordance with the principles and procedures approved by the Committee on the Ethics of Animal Experiments of Anhui University of Science and Technology (Anhui, People’s Republic of China). To facilitate the injection of experimental drugs, measurement of tumor volume, weighing the mice, and taking pictures of the mice, we anesthetized them before the following experimental operations with sodium pentobarbital, which is formulated into 1 to 3% physiological saline solution and administered by 30 mg/kg body weight by intraperitoneal injection. Five-seven-7-week-old female BALB/c-nu/nu nude mice were selected to xenograft studies. First, suspensions of 5 × 10^7^/0.2 mL SK-Hep1 cells were injected subcutaneously into the right hindlimbs. When tumor volumes reached 200 mm^3^, one treated group received 25 mg/kg PKI-587 every four days via the tail vein; one treated group received 2 Gy IR every other day for four treatments; one treated groups received 25 mg/kg PKI-587 combined with IR every four days, and in combination therapy, PKI-587 was administered 6 h before IR exposure. We used the formula (length × width^2^)/2 to calculate tumor size every 2 days and recorded the body weights (g).

### Immunohistochemistry assays

Five-micrometer tissue sections harvested from the SK-Hep1 xenografts were used for immunohistochemical (IHC) staining. The sections were fixed with 4% formaldehyde for 24 h, embedded with paraffin, incubated at 4°C with anti-Ki67 antibodies (1:600 dilution) for 24 h, and incubated with secondary antibodies (SP-9000) for 60 min. The slides were counterstained with hematoxylin and eosin and observed with a microscope.

### Statistical analysis

All experiments were repeated three times independently, and two-tailed unpaired Student t test was used to analyze data in in vitro experiment, and two-way ANOVA followed by multiple comparison test was used to analyze data in in vivo experiment. *P* <0.05 was taken as statistically significant.

## Results

### PKI-587 blocked PI3K/AKT/mTOR induced by radiation and inhibited DNA damage repair pathway when combined with radiation

**Fig 1:**
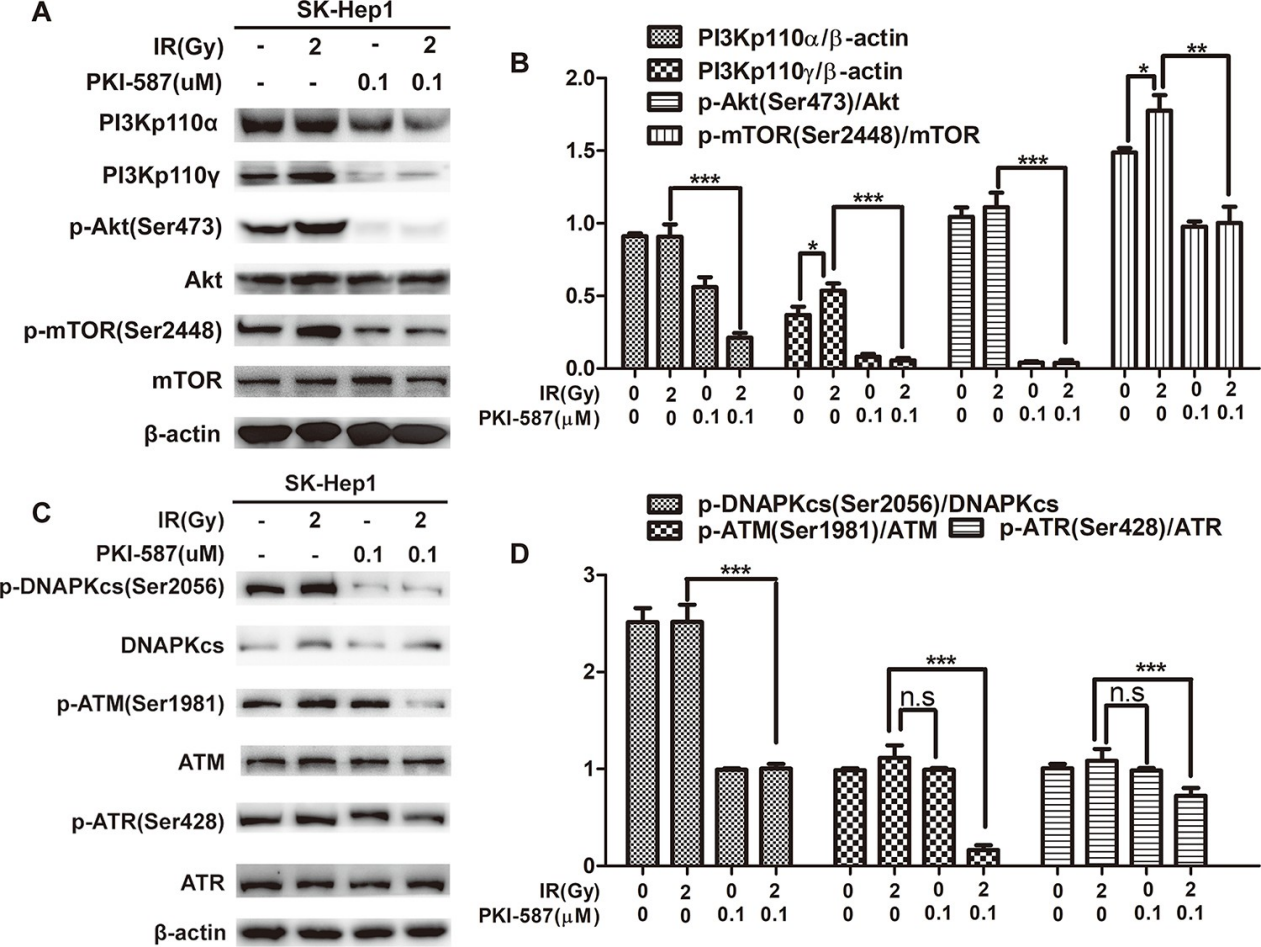
PKI-587 blocked PI3K/AKT/mTOR induced by radiation and inhibited the DNA damage repair pathway when combined with radiation. (A and B) Western blot assay and semiquantitative analysis of the levels of PI3Kp110α, PI3Kp110γ, p-Akt (Ser473), Akt, p-mTOR (Ser2448), and mTOR proteins involved in PI3K/AKT/mTOR pathway in SK-Hep1 cells after radiation and PKI-587 alone or in combination treatment for 24 h. (C and D) Western blot assay and semiquantitative analysis revealed the levels of related proteins in DNA damage repair pathway, including p-DNAPKcs (Ser2056), DNAPKcs, p-ATR (Ser428), ATR, p-ATM (Ser1981), and ATM in SK-Hep1 cells after radiation and PKI-587 alone or in combination treatment for 24 h. The data are mean ± SD, n = 3. *P<0.05, **P<0.01, ***P<0.001; n.s: no significance. versus IR group. IR: ionizing radiation (6MV-X ray).

To explore the effect of radiation on PI3K/Akt/mTOR and DNA damage repair pathways (NHEJ and HR), we first examined the activation levels of PI3Kp110α, PI3Kp110γ, Akt, mTOR, DNAPKcs, ATM, and ATR in SK-Hep1 cells. Western blot revealed that PI3Kp110γ and p-mTOR (Ser2448) were increased in SK-Hep1 cells after radiation treatment (Fig 1). This indicated that the PI3K/AKT/mTOR signaling pathway in SK-Hep1 cells was further activated after being stimulated by radiation, and PKI-587 inhibited PI3Kp110γ and p-mTOR induced by radiation, as well as PI3Kp110α, p-Akt. At the same time, we found that PKI587 combined with radiation inhibited the DNA damage repair pathway, especially the activity of HR-related kinase ATM and ATR (Fig 1). These results indicated that PKI-587 blocked the abnormal activation of PI3K/Akt/mTOR induced by radiation and inhibited the DNA damage repair pathway when combined with radiation.

### PKI-587 increased the radiosensitivity of HCC cells by inhibiting proliferation

**Fig 2:**
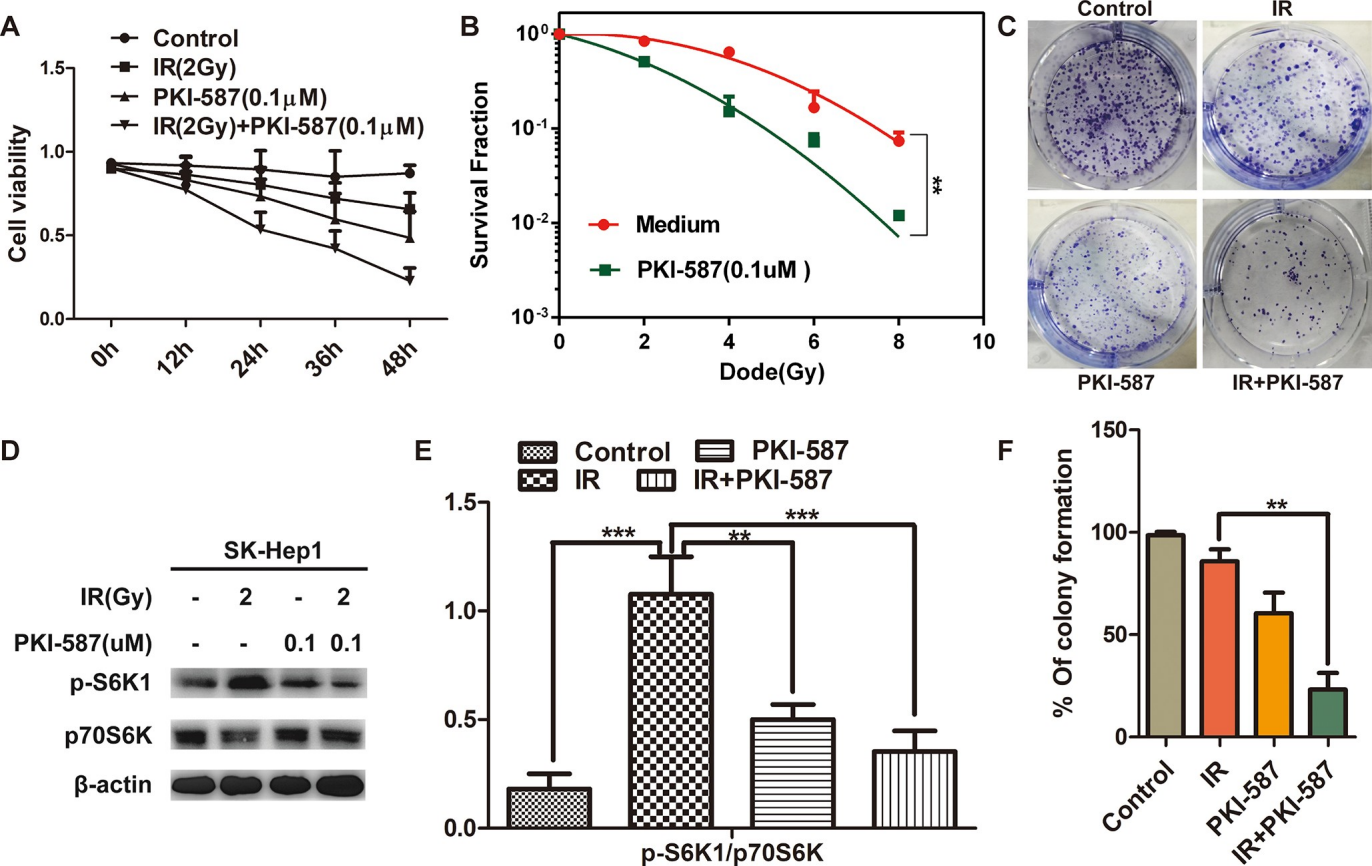
PKI-587 increased the radiosensitivity of HCC cells by inhibiting proliferation. (A) Cytotoxicity of SK-Hep1 cells treated with IR (2Gy) alone or combined with PKI-587 (0.1 μM) at 0, 12, 24, 36, and 48 h. (B) Clonogenic survival assays of SK-Hep1 cells treated with PKI-587 (0.1μM) followed by irradiation in a range of radiation doses. (C and F) Representative images of the colony formation revealing the proliferation of SK-Kep1 cells treated with IR (2 Gy) alone or combined with PKI-587 (0.1 μM) for 24 h. The percentage of colony formation was calculated. (D and E) The level of p-S6K1 and p70S6K proteins in SK-Hep1 cells after treatment with IR (2Gy) alone or combined with PKI-587 (0.1 μM) for 24 h determined by western blot assay. The semiquantitative data were represented as p-S6K1/p70S6K. The data are mean ± SD, n = 3. **P<0.01, ***P<0.001 versus IR group. IR, ionizing radiation (6 MV-X ray).

To explore whether PKI-587 inhibited the abnormal activation of the PI3K/Akt/mTOR signaling pathway while enhancing the radiosensitivity of HCC cells, we evaluated the effect of PKI-587 on proliferation of SK-Hep1 cells by MTT assay. As illustrated in Fig 2A, PKI-587 combined with radiation inhibited the proliferation of SK-Hep1 cells more than did PKI-587 or radiation alone, and it did so in a time-dependent manner. As expected, the combination of IR with PKI-587 reduced the survival fraction post-IR in SK-Hep1 cells (Fig 2B) (*P <* 0.01). The results of colony formation assay revealed that the number of clones was significantly less with PKI-587 combined with radiation than with radiation treatment alone, (Fig 2C and 2F) (*P <* 0.01). We also explored, through western blot experiments, whether the proliferation/survival downstream effector molecule p70S6K, eIF4EBP1 in mTOR was inhibited by PKI-587 treatment. As illustrated in Fig 2D and 2E, radiation treatment increased the level of phosphorylated p70S6K (p-S6K1), whereas p-S6K1 was decreased by PKI-587 treatment, and it was further decreased with combined PKI-587 and radiation treatment. Additionally, compared to radiation alone, the combination of IR with PKI-587 reduced the level of phosphorylation of eIF4EBP1 (S1 Fig). These results indicated that PKI-587 combined with radiation inhibited the proliferation of HCC cells by suppressing the phosphorylation of p70S6K and eIF4EBP1, the downstream effector molecule of the PI3K/Akt/mTOR signaling pathway.

### Persistence of γ-H2AX foci suggested that PKI-587 increased radiosensitization of HCC cells

**Fig 3:**
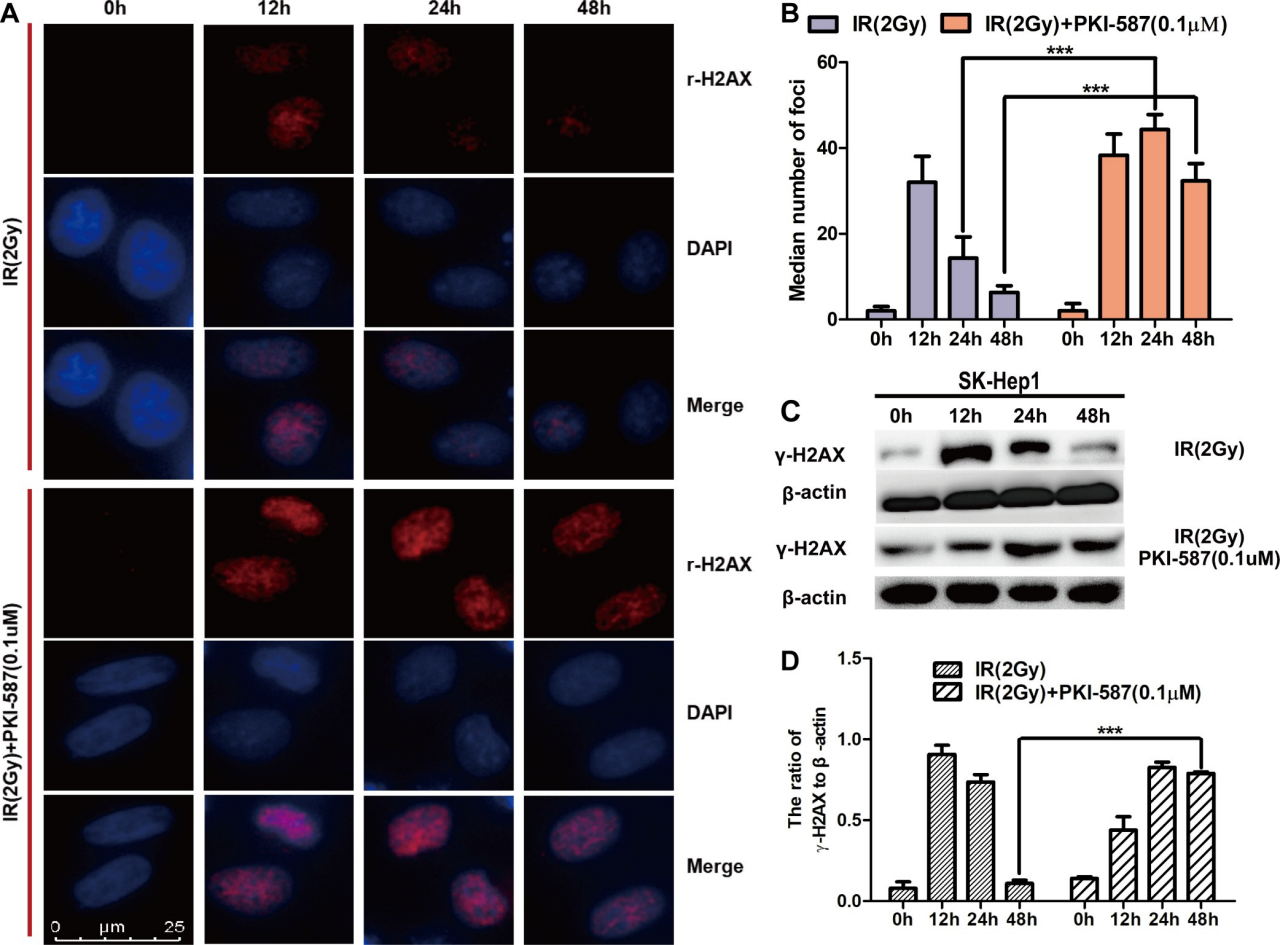
PKI-587 induced the persistence of γ-H2AX foci. (A and B) Representative immunofluorescence micrographs (400x) of γ-H2AX foci formation with IR (2 Gy) alone or combined with PKI-587 (0.1 μM) at 0, 12, 24, and 48 h. ***P<0.001 versus 24 h or 48 h group. (C and D) Western blotting analysis of γ-H2AX level in SK-Hep1 cells treated with IR (2 Gy) alone or combined with PKI-587 (0.1 μM) at 0, 12, 24, and 48 h. The data are mean ± SD, n = 3. ***P<0.001 versus 48 h group. IR, ionizing radiation (6 MV-X ray).

To explore the effect of PKI-587 on radiation-induced DNA double-strand breaks, we first assessed, by indirect immunofluorescence assay, changes in the number of γ-H2AX foci in the nucleus after treatment with IR (2 Gy) alone or combined with PKI-587 (0.1 μM) for 0, 12, 24, and 48 h. The results revealed that the number of γ-H2AX foci was maximum after treatment with radiation alone for 12 h, then gradually decreased. PKI-587 combined with radiation significantly increased the formation of γ-H2AX foci compared with that of radiation treatment alone, especially at 24 h (Fig 3A and 3B). Correspondingly, Western blot analysis revealed that the expression level of γ-H2AX protein reached a peak after radiation treatment for 12 h, then gradually decreased; the expression level of γ-H2AX increased significantly also after treatment with PKI-587 combined with radiation for 24 h (Fig 3C and 3D). These results indicated that PKI-587 combined with radiation induces the persistent presence of DNA breakpoints and high level of γ-H2AX in SK-Hep1 cells.

### PKI-587 combined with IR promoted cell-cycle arrest in HCC cells

**Fig 4:**
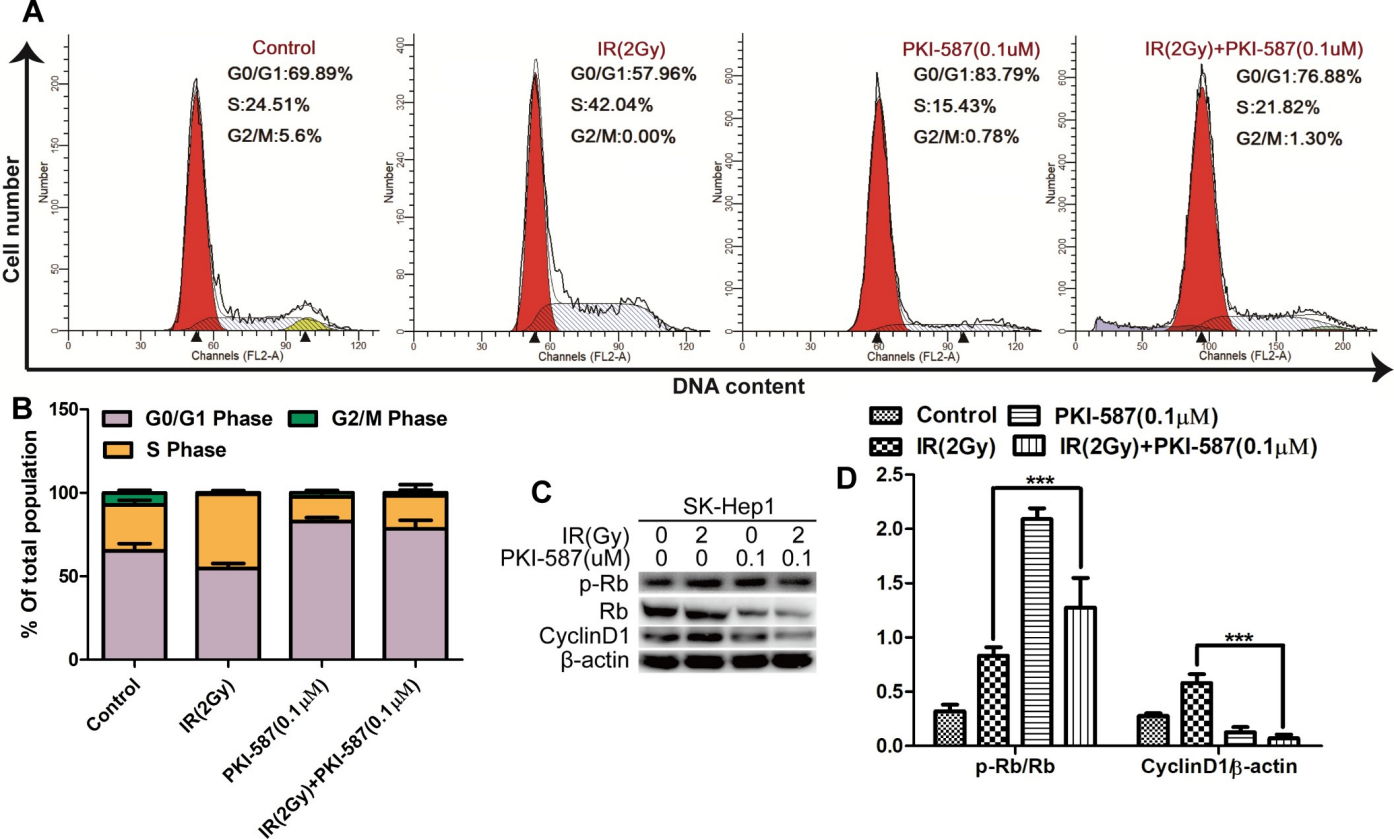
PKI-587 combined with IR promoted G0/G1 phase cell arrest in SK-Hep1 cells. (A and B) Cell-cycle distribution in SK-Hep1 cells as determined with flow cytometry after IR (2 Gy) alone or combined with PKI-587 (0.1 μM) treatment for 24 h. (C and D) Expression of Cyclin D1, Rb and p-Rb in SK-Hep1 cells as determined with western blot analysis. The data are mean ± SD, n = 3. ***P<0.001 versus IR group. IR: ionizing radiation (6MV-X ray).

It has been reported that DNA damage causes changes in the phase distribution of the cell cycle [28, 37]. We monitored, by flow cytometry and propidium iodide staining, the cell cycle of SK-Hep1 cells after treatment with IR (2Gy) alone or combined with PKI-587 (0.1 μM) for 24 h. As illustrated in Fig 4A and 4B, the proportion of S-phase cells in IR-treated cells was 42.1% more than in control cells. Upon treatment with IR (2Gy) combined with PKI-587 (0.1 μM), the proportion of S-phase cells was decreased to 21.8%, and more cells were present in the G0/G1 phase (76.9%), a result that implied that PKI-587 combined with IR blocked cell-cycle progression in the SK-Hep1 cells. We also measured, with western blot assay, p-Rb and CyclinD1 of cells transforming from G0/G1 phase to S phase [28, 38]. Values of p-Rb and CyclinD1 in cells treated with IR (2 Gy) combined with PKI-587 (0.1 μM) were significantly less than in cells treated with IR alone (Fig 4C and 4D). These results are evidence that IR combined with PKI-587 converted the IR-induced SK-Hep1 cells residing in S phase into G0/G1 phase, resulting in increased sensitivity of the cells to IR.

### PKI-587 combined with IR induced apoptosis of HCC cells

**Fig 5:**
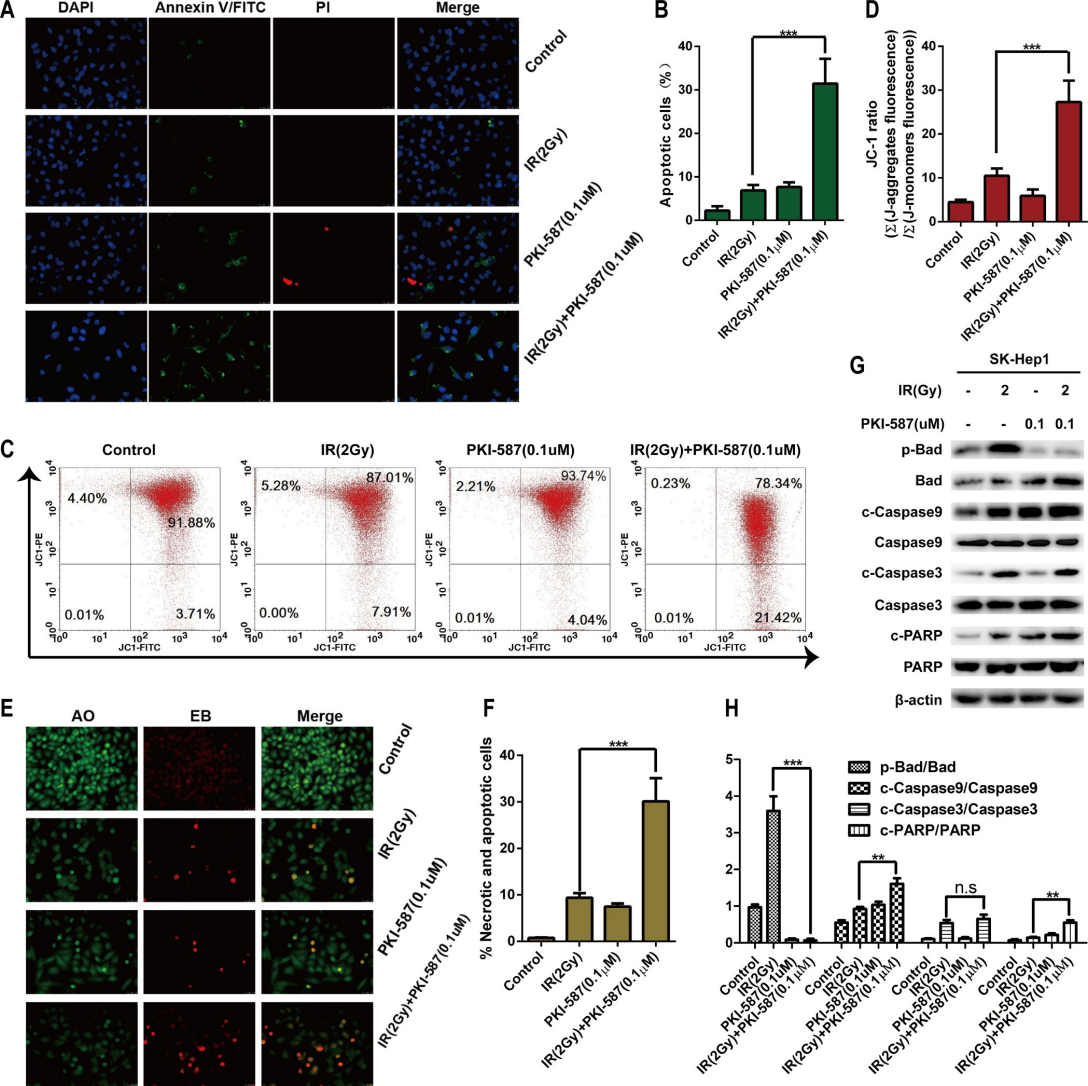
PKI-587 combined with IR effectively induced apoptosis of SK-Hep1 cells. (A and B) Annexin V-FITC/PI/DAPI staining after IR (2Gy) alone or combined with PKI-587 (0.1 μM) treatment for 24 h. Representative images were taken at × 400 magnification. (C and D) Flow cytometry with JC-1 staining after IR (2 Gy) alone or combined with PKI-587 (0.1 μM) treatment for 24 h. (E and F) AO/EB staining after IR (2 Gy) alone or combined with PKI-587 (0.1 μM) treatment for 24 h. Representative images were taken at × 400 magnification. (G and H) Western blot analysis of apoptotic protein after IR (2 Gy) alone or combined with PKI-587 (0.1 μM) treatment for 24 h. The data are mean ± SD, n = 3. n.s, no significance, **P<0.01, ***P<0.001 versus IR group. IR, ionizing radiation (6 MV-X ray).

Activation of the PI3K/AKT/mTOR and DNA damage repair pathways (NHEJ and HR) can inhibit apoptosis of HCC cells [19–23]. Our research has confirmed that PKI-587, when combined with radiation, can block the abnormal activation of PI3K/AKT/mTOR induced by radiation and inhibit the DNA damage repair pathway. We here investigated the effect of PKI-587 combined with radiation on cell apoptosis, as determined with annexin V FITC/PI/DAPI staining. As illustrated in Fig 5A and 5B, compared with IR alone, radiation combined with PKI-587 had a more pro-apoptotic effect on SK-Hep1 cells (*P* < 0.001). Similarly, JC-1 staining for mitochondrial membrane potential revealed a higher rate (21.4%) of apoptosis with PKI-587 combined with radiation treatment than with IR alone (7.91%) (Fig 5C and 5D). AO/EB staining (Fig 5E and 5F) also revealed that PKI-587 combined with radiation exerted a stronger pro-apoptotic effect than did radiation alone. We also evaluated the expression levels of the apoptotic protein molecules p-Bad / Bad, c-Caspase3 / Caspase3 and c-PARP / PARP in western blot experiments. Fig 5G and 5H illustrate that the expression level of pro-apoptotic factor Bad, as well as c-Caspase3 and c-PARP, in PKI-587 combined with radiation was significantly up-regulated, whereas the anti-apoptotic factor p-Bad was suppressed, which indicated that PKI-587 enhanced the pro-apoptotic effect of radiation.

#### PKI-587 combined with radiotherapy can inhibit tumor growth *in vivo*

**Fig 6:**
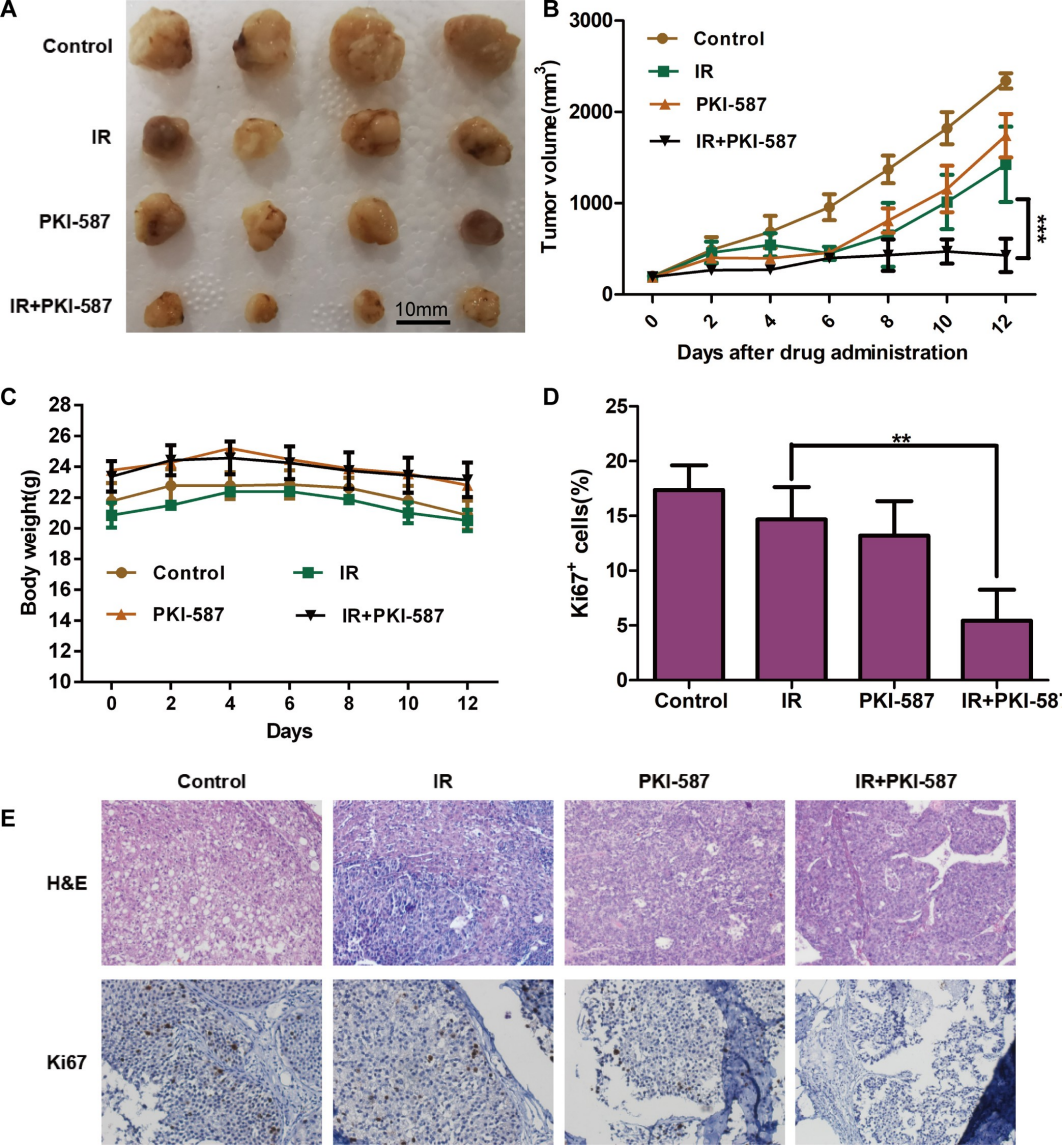
PKI-587 combined with radiotherapy can inhibit tumor growth *in vivo*. (A and B) Antitumor efficacy of PKI-587, IR, and the combination of IR and PKI-587 in SK-Hep1 tumor xenografts by according to xenograft volumes. The data are mean ± SE, n = 4. ***P < 0.001. (C) Mouse body weight of each group during the in vivo experiment. (D and E) Expression of Ki67 assay in the harvested tumors. Representative images were taken at × 400 magnification. Histogram shows percentage of SK-Hep1 cells positive for Ki67. The data are mean ± SD, n = 3. **P < 0.01.

To determine whether PKI-587 combined with radiotherapy can inhibit tumor growth *in vivo*, we used the SK-Hep1 xenograft model. As illustrated in Fig 6A–6C, either PKI-587 or IR alone had antitumor activity, but PKI-587 combined with radiotherapy had a stronger effect, as illustrated by greater reduction in volume of the xenograft (*P* < 0.001). Hematoxylin-eosin staining revealed no significant morphologic abnormalities in any treatment group. The results also showed that significantly fewer Ki67-positive cells were present in the tumors of animals treated with combined IR and PKI-587 than in tumors of animals treated with IR or PKI-587 alone (Fig 6D and 6E). It was well known that Ki67-positive cells represent proliferating cells. The above results indicate that treatment with either IR or PKI-587 slightly inhibited tumor growth, but the combination was more effective. Additionally, we also found that PKI-587 can block PI3K/AKT/mTOR induced by radiation and inhibit DNA damage repair pathway when combined with radiation *in vivo* (S2 Fig). The results are consistent with *in vitro* experiments.

## Discussion

**Fig 7:**
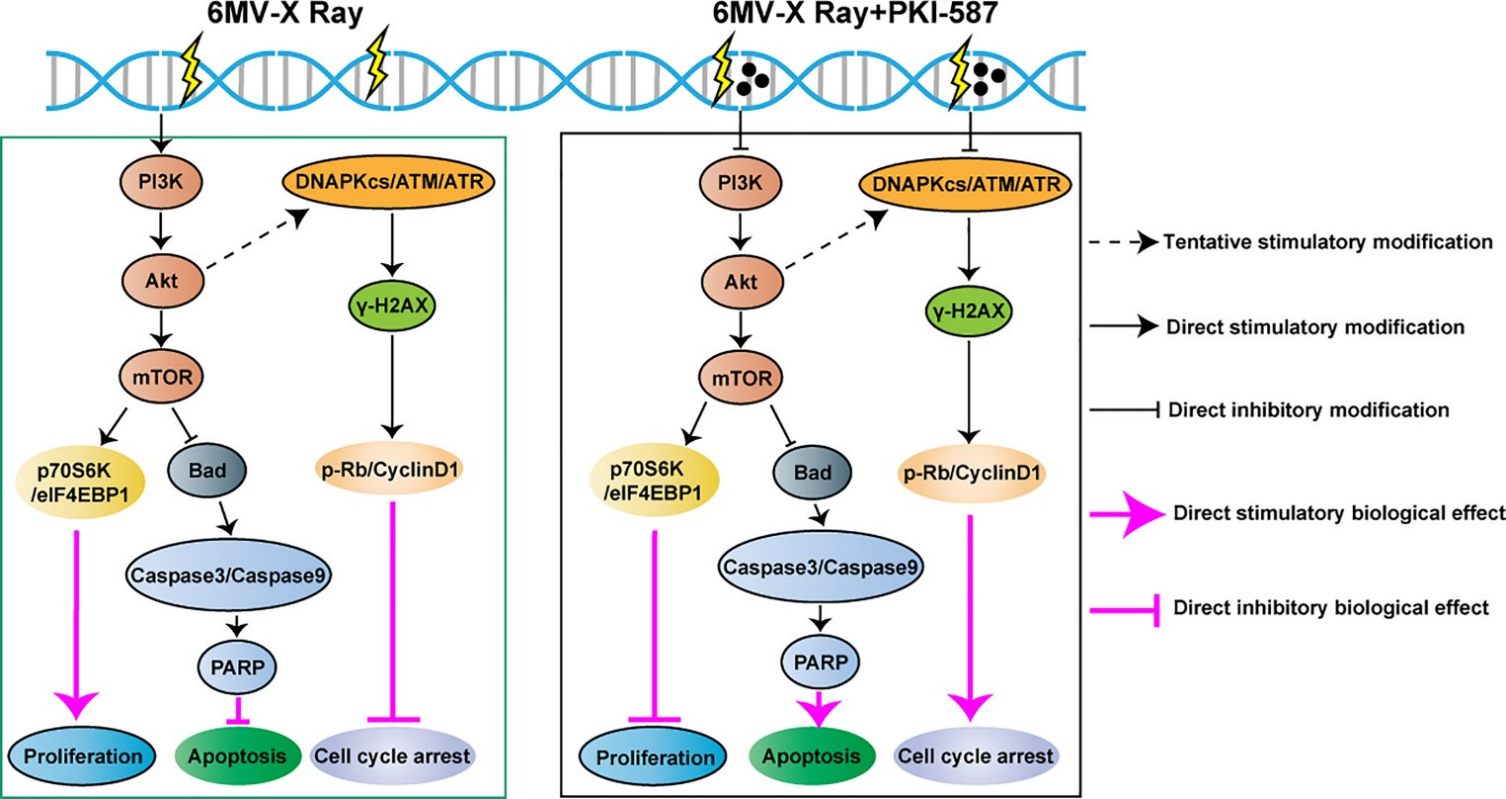
Schematic diagram of IR (6 MV-X ray) inducing resistance of HCC cells and PKI-587 enhancing the radiosensitivity of HCC cells.

We undertook this study to better understand the radioresistance of HCC and ways by which the resistance might be overcome. The major findings were that ionizing radiation induced radiotherapy tolerance in HCC cells by activating the PI3K/AKT/mTOR pathway. The dual inhibitor, PKI-587 inhibited the abnormal activation of this pathway and its downstream effector molecule as well as the DNA damage repair pathway (Fig 7). In in vivo experiments, PKI-587 increased the inhibition of ionizing radiation on tumors in the SK-Hep1 xenograft model.

Radiation mainly induces DNA double-strand breaks, leading to tumor cell damage [39]. After DNA damage occurs, cells initiate DNA damage response to repair the damage. The major repair pathways for DNA double-strand breaks are homologous recombination (HR) and non-homologous end joining (NHEJ) [40]. In this work, we did not find that the DNA damage repair pathway of HCC cells was significantly activated after radiation. However, PKI-587 alone or in combination with radiation reduced the phosphorylation of DNAPKcs in non-homologous NHEJ repair, and PKI-587 significantly suppressed the activation of the homologous recombination repair kinase ATM and ATR when combined with radiation. Correspondingly, the expression levels of γ-H2AX, P-Rb, Rb and CyclinD1 were reduced, resulting in pro-apoptosis and anti-proliferation of cells.

The desensitization of HCC to radiotherapy is due to the radiation-induced abnormal activation of the PI3K/AKT/mTOR signaling pathway [22, 41]. The interaction of AKT1, AKT3, and DNA-PKcs promote tumor-cell proliferation and survival after irradiation [42]. AKT1 regulates HR repair of DNA broken double-strands in a Rad51-dependent manner, and inhibition of AKT kinase activity reduces the Rad51 protein and Rad51 foci formation and inhibits homologous recombination repair [43, 44]. These observations indicate that there may be cross talk between PI3K/AKT/mTOR and DNA damage repair pathways. Previous studies have found that drugs can activate the PI3K/AKT/mTOR and DNA damage repair pathways, and the combined application of PKI-587 can inhibit the activation of these two pathways, thereby enhancing the sensitivity of tumor cells to drugs [28]. This study found that radiation only activates the PI3K/AKT/mTOR pathway, but the combined application of PKI-587 can simultaneously inhibit PI3K/AKT/mTOR and DNA damage repair pathways, thereby enhancing the sensitivity of tumor cells to radiation, which may involve complex signaling pathway networks. In this study, the radiation failure to directly activate DNA damage repair pathway may be related to the negative regulation of AKT. Due to the impact of the COVID-19, we have not been able to conduct in-depth research on this issue, which is a new discovery that deserves further evaluation.

The PI3K/AKT/mTOR signaling pathway in tumor cells is further activated by radiation, resulting in tumorigenesis, progression, angiogenesis, invasion, metastasis, and anti-apoptosis [45, 46]. The mTOR downstream effector molecule, S6 protein kinase 1 (S6K1), especially regulates cellular protein translation. Phosphorylated S6K1 activates the corresponding ribosomal S6k protein, which promotes the translation of mRNA and synthesis of ribosomal protein [47, 48]. Our results demonstrated that PKI-587 combined with radiation can reduce the phosphorylation level of S6K1 induced by radiation, thereby inhibiting the proliferation of HCC cells and enhancing radiotherapy efficacy.

Radiation induces the cascade activation of PI3K/AKT/mTOR signaling pathway and downstream molecule S6K to regulate the mitochondrial apoptosis pathway by down-regulating Bad expression. Bad is induced by calmodulin to dephosphorylate and undergo a conformational change, translocating to the outer mitochondrial membrane and replacing mitochondrial anti-apoptosis. Bax on the outer mitochondrial membrane is bound by the protein Bcl-xL and dissociated to change the membrane permeability, leading to release of cytochrome C outside the mitochondria. The cytochrome C cascade activates caspase-9, caspase-3, caspase-7, and PARP and induces apoptosis [49, 50]. Our research established that radiation combined with PKI-587 inhibited the PI3K/AKT/mTOR signaling pathway, down-regulated phosphorylated Bad, and increased mitochondrial apoptosis in HCC cells. The detailed mechanism will be examined in our future research.

Our in vitro experiments documented convincingly that PKI-587 down-regulates the activities of the PI3K/AKT/mTOR and DNA damage repair pathways in HCC cells, weakening the radiation resistance and increasing the radiosensitization of cells. In vivo experiments extended those results by disclosing that the addition of PKI-587 to IR increased the anti-tumor effect of IR in the xenograft HCC model. However, because of the complexity of the tumor microenvironment, we cannot be certain whether PKI-587 acted through down-regulation of these two pathways or by other mechanisms. More in vivo experiments will be needed to clarify the molecular events in the effects of PKI-587 combined with radiation on HCC.

## Conclusion

PKI-587 inhibited proliferation, promoted apoptosis, and enhanced radiosensitization of hepatocellular carcinoma cells by blocking abnormal, radiation-induced activation of the PI3K/AKT/mTOR and DNA damage repair pathways and their downstream effector molecules. The addition of PKI-587 to IR increased the anti-tumor effect of radiation in the xenograft HCC model. These findings support the possibility that the combination of radiation and PKI-587 can be an effective strategy for treatment of hepatocellular carcinoma.

## Supporting information

S1 Checklist ARRIVE guidelines 2.0 checklist. A copy of the Full ARRIVE 2.0 Guidelines checklist, a document that aims to improve experimental reporting and reproducibility of animal studies for purposes of post-publication data analysis and reproducibility: https://arriveguidelines.org/sites/arrive/files/Author%20Checklist%20-%20Full.pdf (PDF).
(DOC)

S1 Fig PKI-587 increased the radiosensitivity of HCC cells by inhibiting proliferation. (A and B) The level of p-eIF4EBP1 and eIF4EBP1 proteins in SK-Hep1 cells after treatment with IR (2Gy) alone or combined with PKI-587 (0.1 μM) for 24 h as determined by western blot assay. The semiquantitative data were represented as p-eIF4EBP1/eIF4EBP1. The data are mean ± SD, n = 3. ***P<0.001. IR, ionizing radiation (6 MV-X ray).
(TIF)

S2 Fig PKI-587 blocked PI3K/AKT/mTOR induced by radiation and inhibited DNA damage repair pathway when combined with radiation *in vivo*. (A and B) Western blot assay and semiquantitative analysis of the levels of PI3Kp110α, p-Akt (Ser473), Akt, p-mTOR (Ser2448), and mTOR proteins involved in the PI3K/AKT/mTOR pathway in SK-Hep1 cells after radiation and PKI-587 alone or in combination for 24 h. (C and D) Western blot assay and semiquantitative analysis revealed the levels of related proteins in the DNA damage repair pathway, including p-DNAPKcs (Ser2056), DNAPKcs, p-ATR (Ser428), ATR, p-ATM (Ser1981), and ATM in SK-Hep1 cells after radiation and PKI-587 alone or in combination for 24 h. The data are mean ± SD, n = 3. *P<0.05, ***P<0.001. IR: ionizing radiation (6MV-X ray).
(TIF)

S1 Raw images The original uncropped and unadjusted images underlying all blot or gel results reported in a submission’s figures or S1 File. (PDF)

S1 File The minimal underlying data set. (ZIP)

## References

[1] Bray F, Ferlay J, Soerjomataram I, Siegel RL, Torre LA, Jemal A. Global cancer statistics 2018: GLOBOCAN estimates of incidence and mortality worldwide for 36 cancers in 185 countries. CA Cancer J Clin. 2018;68: 394–424. doi: 10.3322/caac.21492 30207593

[2] Zhou M, Wang H, Zeng X, Yin P, Zhu J, Chen W, et al. Mortality, morbidity, and risk factors in China and its provinces, 1990–2017: a systematic analysis for the Global Burden of Disease Study 2017. Lancet. 2019;394: 1145–1158. doi: 10.1016/S0140-6736(19)30427-1 31248666 PMC6891889

[3] Gan H, Chen L, Sui X, Wu B, Zou S, Li A, et al. Enhanced delivery of sorafenib with anti-GPC3 antibody-conjugated TPGS-b-PCL/Pluronic P123 polymeric nanoparticles for targeted therapy of hepatocellular carcinoma. Mater Sci Eng C Mater Biol Appl. 2018;91: 395–403. doi: 10.1016/j.msec.2018.05.011 30033270

[4] Tang X, Lyu Y, Xie D, Li A, Liang Y, Zheng D. Therapeutic Effect of Sorafenib-Loaded TPGS-*b*-PCL Nanoparticles on Liver Cancer. J Biomed Nanotechnol. 2018;14: 396–403. doi: 10.1166/jbn.2018.2529 31352936

[5] Tang X, Li Q, Zhu Y, Zheng D, Dai J, Ni W, et al. The advantages of PD1 activating chimeric receptor (PD1-ACR) engineered lymphocytes for PDL1(+) cancer therapy. Am J Transl Res. 2015;7: 460–73. 26045887 PMC4448187

[6] Crocetti L, Bargellini I, Cioni R. Loco-regional treatment of HCC: current status. Clin Radiol. 2017;72: 626–635. doi: 10.1016/j.crad.2017.01.013 28258743

[7] Facciorusso A, Di Maso M, Muscatiello N. Microwave ablation versus radiofrequency ablation for the treatment of hepatocellular carcinoma: A systematic review and meta-analysis. Int J Hyperthermia. 2016;32: 339–344. doi: 10.3109/02656736.2015.1127434 26794414

[8] Sangiovanni A, Colombo M. Treatment of hepatocellular carcinoma: beyond international guidelines. Liver Int. 2016;36: 124–129. doi: 10.1111/liv.13028 26725909

[9] Wang J, Zhao H, Yu J, Xu X, Liu W, Jing H, et al. MiR-92b targets p57kip2 to modulate the resistance of hepatocellular carcinoma (HCC) to ionizing radiation (IR) -based radiotherapy. Biomed Pharmacother. 2019;110: 646–655. doi: 10.1016/j.biopha.2018.11.080 30544064

[10] Yoon HI, Seong J. Optimal Selection of Radiotherapy as Part of a Multimodal Approach for Hepatocellular Carcinoma. Liver Cancer. 2016;5: 139–151. doi: 10.1159/000367762 27386432 PMC4906424

[11] Choi SH, Seong J. Strategic application of radiotherapy for hepatocellular carcinoma. Clin Mol Hepatol. 2018;24: 114–134. doi: 10.3350/cmh.2017.0073 29439305 PMC6038936

[12] Teraoka Y, Kimura T, Aikata H, Daijo K, Osawa M, Honda F, et al. Clinical outcomes of stereotactic body radiotherapy for elderly patients with hepatocellular carcinoma. Hepatol Res. 2018;48: 193–204. doi: 10.1111/hepr.12916 28544062

[13] Eggert T, Greten TF. Current Standard and Future Perspectives in Non-Surgical Therapy for Hepatocellular Carcinoma. Digestion. 2017;96: 1–4. doi: 10.1159/000464282 28605745 PMC5548590

[14] Chino F, Stephens SJ, Choi SS, Marin D, Kim CY, Morse MA, et al. The role of external beam radiotherapy in the treatment of hepatocellular cancer. Cancer. 2018;124: 3476–3489. doi: 10.1002/cncr.31334 29645076

[15] Wahl DR, Stenmark MH, Tao Y, Pollom EL, Caoili EM, Lawrence TS, et al. Outcomes After Stereotactic Body Radiotherapy or Radiofrequency Ablation for Hepatocellular Carcinoma. J Clin Oncol. 2016;34: 452–459. doi: 10.1200/JCO.2015.61.4925 26628466 PMC4872011

[16] Wada Y, Takami Y, Matsushima H, Tateishi M, Ryu T, Yoshitomi M, et al. The Safety and Efficacy of Combination Therapy of Sorafenib and Radiotherapy for Advanced Hepatocellular Carcinoma: A Retrospective Study. Intern Med. 2018;57: 1345–1353. doi: 10.2169/internalmedicine.9826-17 29279513 PMC5995712

[17] Chen YH, Wang CW, Wei MF, Tzeng YS, Lan KH, Cheng AL, et al. Maintenance BEZ235 Treatment Prolongs the Therapeutic Effect of the Combination of BEZ235 and Radiotherapy for Colorectal Cancer. Cancers (Basel). 2019;11: 1204. doi: 10.3390/cancers11081204 31430901 PMC6721476

[18] Fatehi D, Soltani A, Ghatrehsamani M. SRT1720, a potential sensitizer for radiotherapy and cytotoxicity effects of NVB-BEZ235 in metastatic breast cancer cells. Pathol Res Pract. 2018;214: 889–895. doi: 10.1016/j.prp.2018.04.001 29653746

[19] Yu CC, Hung SK, Lin HY, Chiou WY, Lee MS, Liao HF, et al. Targeting the PI3K/AKT/mTOR signaling pathway as an effectively radiosensitizing strategy for treating human oral squamous cell carcinoma *in vitro* and *in vivo*. Oncotarget. 2017;8: 68641–68653. doi: 10.18632/oncotarget.19817 28978144 PMC5620284

[20] Liu Y, Shen Y, Sun T, Yang W. Mechanisms regulating radiosensitivity of glioma stem cells. Neoplasma. 2017;64: 655–665. doi: 10.4149/neo_2017_502 28592117

[21] Han MW, Ryu IS, Lee JC, Kim SH, Chang HW, Lee YS, et al. Phosphorylation of PI3K regulatory subunit p85 contributes to resistance against PI3K inhibitors in radioresistant head and neck cancer. Oral Oncol. 2018;78: 56–63. doi: 10.1016/j.oraloncology.2018.01.014 29496059

[22] Bamodu OA, Chang HL, Ong JR, Lee WH, Yeh CT, Tsai JT. Elevated PDK1 Expression Drives PI3K/AKT/MTOR Signaling Promotes Radiation-Resistant and Dedifferentiated Phenotype of Hepatocellular Carcinoma. Cells. 2020;9: 746. doi: 10.3390/cells9030746 32197467 PMC7140693

[23] Aoki M, Fujishita T. Oncogenic Roles of the PI3K/AKT/mTOR Axis. Curr Top Microbiol Immunol. 2017;407: 153–189. doi: 10.1007/82_2017_6 28550454

[24] Huang RX, Zhou PK. DNA damage response signaling pathways and targets for radiotherapy sensitization in cancer. Signal Transduct Target Ther. 2020;5(1):60. doi: 10.1038/s41392-020-0150-x 32355263 PMC7192953

[25] Sage E, Shikazono N. Radiation-induced clustered DNA lesions: Repair and mutagenesis. Free Radic Biol Med. 2017;107: 125–135. doi: 10.1016/j.freeradbiomed.2016.12.008 27939934

[26] Chatterjee N, Walker GC. Mechanisms of DNA damage, repair, and mutagenesis. Environ Mol Mutagen. 2017;58: 235–263. doi: 10.1002/em.22087 28485537 PMC5474181

[27] Karimian A, Mir SM, Parsian H, Refieyan S, Mirza-Aghazadeh-Attari M, Yousefi B, et al. Crosstalk between Phosphoinositide 3-kinase/Akt signaling pathway with DNA damage response and oxidative stress in cancer. J Cell Biochem. 2019;120: 10248–10272. doi: 10.1002/jcb.28309 30592328

[28] Zhang Y, Xie C, Li A, Liu X, Xing Y, Shen J, et al. PKI-587 enhances chemosensitivity of oxaliplatin in hepatocellular carcinoma through suppressing DNA damage repair pathway (NHEJ and HR) and PI3K/AKT/mTOR pathway. Am J Transl Res. 2019;11: 5134–5149. 31497229 PMC6731445

[29] Miyahara H, Yadavilli S, Natsumeda M, Rubens JA, Rodgers L, Kambhampati M, et al. The dual mTOR kinase inhibitor TAK228 inhibits tumorigenicity and enhances radiosensitization in diffuse intrinsic pontine glioma. Cancer Lett. 2017; 400: 110–116. doi: 10.1016/j.canlet.2017.04.019 28450157 PMC5569904

[30] Prêtre V, Wicki A. Inhibition of Akt and other AGC kinases: A target for clinical cancer therapy? Semin Cancer Biol. 2018;48: 70–77. doi: 10.1016/j.semcancer.2017.04.011 28473255

[31] Shi JJ, Xing H, Wang YX, Zhang X, Zhan QM, Geng MY, et al. PI3Kα inhibitors sensitize esophageal squamous cell carcinoma to radiation by abrogating survival signals in tumor cells and tumor microenvironment. Cancer Lett. 2019;459: 145–155. doi: 10.1016/j.canlet.2019.05.040 31173854

[32] Li A, Zhang R, Zhang Y, Liu X, Wang R, Liu J, et al. BEZ235 increases sorafenib inhibition of hepatocellular carcinoma cells by suppressing the PI3K/AKT/mTOR pathway. Am J Transl Res. 2019;11: 5573–5585. 31632530 PMC6789287

[33] Del Campo JM, Birrer M, Davis C, Fujiwara K, Gollerkeri A, Gore M, et al. A randomized phase II non-comparative study of PF-04691502 and gedatolisib (PF-05212384) in patients with recurrent endometrial cancer. Gynecol Oncol. 2016;142: 62–69. doi: 10.1016/j.ygyno.2016.04.019 27103175

[34] Freitag H, Christen F, Lewens F, Grass I, Briest F, Iwaszkiewicz S, et al. Inhibition of mTOR’s Catalytic Site by PKI-587 Is a Promising Therapeutic Option for Gastroenteropancreatic Neuroendocrine Tumor Disease. Neuroendocrinology. 2017;105: 90–104. doi: 10.1159/000448843 27513674 PMC5475233

[35] Leiker AJ, DeGraff W, Choudhuri R, Sowers AL, Thetford A, Cook JA, et al. Radiation Enhancement of Head and Neck Squamous Cell Carcinoma by the Dual PI3K/mTOR Inhibitor PF-05212384. Clin Cancer Res. 2015;21: 2792–801. doi: 10.1158/1078-0432.CCR-14-3279 25724523 PMC4470749

[36] Liu T, Li Q, Sun Q, Zhang Y, Yang H, Wang R, et al. MET inhibitor PHA-665752 suppresses the hepatocyte growth factor-induced cell proliferation and radioresistance in nasopharyngeal carcinoma cells. Biochem Biophys Res Commun. 2014;449: 49–54. doi: 10.1016/j.bbrc.2014.04.147 24802404

[37] Carrassa L, Damia G. DNA damage response inhibitors: Mechanisms and potential applications in cancer therapy. Cancer Treat Rev. 2017;60: 139–151. doi: 10.1016/j.ctrv.2017.08.013 28961555

[38] Murray JM, Carr AM. Integrating DNA damage repair with the cell cycle. Curr Opin Cell Biol. 2018;52: 120–125. doi: 10.1016/j.ceb.2018.03.006 29587168

[39] Lee SY, Jeong EK, Ju MK, Jeon HM, Kim MY, Kim CH, et al. Induction of metastasis, cancer stem cell phenotype, and oncogenic metabolism in cancer cells by ionizing radiation. Mol Cancer. 2017;16: 10. doi: 10.1186/s12943-016-0577-4 28137309 PMC5282724

[40] Du J, Yin N, Xie T, Zheng Y, Xia N, Shang J, et al. Quantitative assessment of HR and NHEJ activities via CRISPR/Cas9-induced oligodeoxynucleotide-mediated DSB repair. DNA Repair (Amst). 2018;70: 67–71. doi: 10.1016/j.dnarep.2018.09.002 30212742

[41] Sheng H, Huang Y, Xiao Y, Zhu Z, Shen M, Zhou P, et al. ATR inhibitor AZD6738 enhances the antitumor activity of radiotherapy and immune checkpoint inhibitors by potentiating the tumor immune microenvironment in hepatocellular carcinoma. J Immunother Cancer. 2020;8: e000340. doi: 10.1136/jitc-2019-000340 32461345 PMC7254123

[42] Toulany M, Maier J, Iida M, Rebholz S, Holler M, Grottke A, et al. Akt1 and Akt3 but not Akt2 through interaction with DNA-PKcs stimulate proliferation and post-irradiation cell survival of K-RAS-mutated cancer cells. Cell Death Discov. 2017;3: 17072. doi: 10.1038/cddiscovery.2017.72 29090098 PMC5661268

[43] Mueck K, Rebholz S, Harati MD, Rodemann HP, Toulany M. Akt1 Stimulates Homologous Recombination Repair of DNA Double-Strand Breaks in a Rad51-Dependent Manner. Int J Mol Sci. 2017;18: 2473. 10.3390/ijms18112473 PMC5713439 29156644

[44] Mohammadian Gol T, Rodemann HP, Dittmann K. Depletion of Akt1 and Akt2 Impairs the Repair of Radiation-Induced DNA Double Strand Breaks via Homologous Recombination. Int J Mol Sci. 2019;20: 6316. doi: 10.3390/ijms20246316 31847370 PMC6941063

[45] Toulany M, Rodemann HP. Phosphatidylinositol 3-kinase/Akt signaling as a key mediator of tumor cell responsiveness to radiation. Semin Cancer Biol. 2015;35: 180–90. doi: 10.1016/j.semcancer.2015.07.003 26192967

[46] Saxton RA, Sabatini DM. mTOR Signaling in Growth, Metabolism, and Disease. Cell. 2017;168: 960–976. doi: 10.1016/j.cell.2017.02.004 28283069 PMC5394987

[47] Wang L, Rhodes CJ, Lawrence JC Jr. Activation of mammalian target of rapamycin (mTOR) by insulin is associated with stimulation of 4EBP1 binding to dimeric mTOR complex 1. J Biol Chem. 2006;281: 24293–303. doi: 10.1074/jbc.M603566200 16798736

[48] Hua H, Kong Q, Zhang H, Wang J, Luo T, Jiang Y. Targeting mTOR for cancer therapy. J Hematol Oncol. 2019;12: 71. doi: 10.1186/s13045-019-0754-1 31277692 PMC6612215

[49] Yao A, Shen Y, Wang A, Chen S, Zhang H, Chen F, et al. Sulforaphane induces apoptosis in adipocytes via Akt/p70s6k1/Bad inhibition and ERK activation. Biochem Biophys Res Commun. 2015;465: 696–701. doi: 10.1016/j.bbrc.2015.08.049 26296464

[50] Zhang Z, Zhang H, Chen S, Xu Y, Yao A, Liao Q, et al. Dihydromyricetin induces mitochondria-mediated apoptosis in HepG2 cells through down-regulation of the Akt/Bad pathway. Nutr Res. 2017;38: 27–33. doi: 10.1016/j.nutres.2017.01.003 28381351

