# Supplementary material for: PKI-587 enhances radiosensitization of hepatocellular carcinoma by inhibiting the PI3K/AKT/mTOR pathways and DNA damage repair
Source: PLoS One. 2021 Oct 19;16(10):e0258817. doi: 10.1371/journal.pone.0258817 (PMC8525768; doi:10.1371/journal.pone.0258817)
Supplement: S1 Checklist — A copy of the Full ARRIVE 2.0 Guidelines checklist, a document that aims to improve experimental reporting and reproducibility of animal studies for purposes of post-publication data analysis and reproducibility: https://arriveguidelines.org/sites/arrive/files/Author%20Checklist%20-%20Full.pdf (PDF). (DOC) [file pone.0258817.s001.doc]

**The ARRIVE Essential 10**

**Study design**

1 For each experiment, provide brief details of study design including:

a. The groups being compared, including control groups. If no control group has been used, the

rationale should be stated.

Response: Yes, the groups being compared, including control groups.

b. The experimental unit (e.g. a single animal, litter, or cage of animals).

Response: The experimental unit is a single animal.

**Sample size**

2

a. Specify the exact number of experimental units allocated to each group, and the total number

in each experiment. Also indicate the total number of animals used.

Response: We used 20 mice in total, of which 16 mice were successfully transplanted with tumors. Each group had four mice.

b. Explain how the sample size was decided. Provide details of any a priori sample size

calculation, if done.

Response: Tumor volume was measured with a caliper two perpendicular tumor diameters every two days, and the formula [(length)×(width)2/2] was used to estimate the tumor growth.

**Inclusion and exclusion criteria**

3

a. Describe any criteria used for including and excluding animals (or experimental units) during

the experiment, and data points during the analysis. Specify if these criteria were established

a priori. If no criteria were set, state this explicitly.

Response: Mice that failed to take the tumor or died during the trial were excluded, a criteria that was established before the experiment began.

b. For each experimental group, report any animals, experimental units or data points not

included in the analysis and explain why. If there were no exclusions, state so.

Response: We used 20 mice in total, of which 16 mice were successfully transplanted with tumors. No mice died accidentally during the experiment. So, there were 4 mice excluded in the analysis.

c. For each analysis, report the exact value of n in each experimental group.

Response: We used 16 mice and divided them into four groups. Each group had four mice (n=4).

**Randomisation**

4

a. State whether randomisation was used to allocate experimental units to control and

treatment groups. If done, provide the method used to generate the randomisation sequence. Response: Yes, randomisation was used to allocate experimental units to control and

treatment groups. We use a random number table for complete randomization of groups.

b. Describe the strategy used to minimise potential confounders such as the order of

treatments and measurements, or animal/cage location. If confounders were not controlled,

state this explicitly.

Response: The mice in each group were also treated or measured in a random catch manner.

**Blinding**

5

Describe who was aware of the group allocation at the different stages of the experiment (during

the allocation, the conduct of the experiment, the outcome assessment, and the data analysis). Response: Different operators are responsible for different stages of the experiment, and no information is shared among the operators.

**Outcome measures**

6

a. Clearly define all outcome measures assessed (e.g. cell death, molecular markers, or

behavioural changes).

Response: Tumor size and body weight of the mice were assessed.

b. For hypothesis-testing studies, specify the primary outcome measure, i.e. the outcome

measure that was used to determine the sample size.

Response: Tumor size of the mice was the primary outcome measure.

**Statistical methods**

7

a. Provide details of the statistical methods used for each analysis, including software used. Response: The data were analysed by two-way ANOVA using GraphPad Prism 5 (GraphPad Software, Inc., San Diego, CA, USA). P <0.05 was taken as statistically significant.

b. Describe any methods used to assess whether the data met the assumptions of the

statistical approach, and what was done if the assumptions were not met.

Response: The parametric method was used to assess whether the data met the assumptions of the statistical approach, and if not, the nonparametric method was used.

**Experimental animals**

8

a. Provide species-appropriate details of the animals used, including species, strain and

substrain, sex, age or developmental stage, and, if relevant, weight.

Response: 5-7-week-old female BALB/c-nu/nu nude mice were used in the experiment.

b. Provide further relevant information on the provenance of animals, health/immune status,

genetic modification status, genotype, and any previous procedures.

Response:

The provenance of animals: Changzhou Cavins Laboratory Animal Co., Ltd.;

Health/immune status: Health/Immune system deficiencygenetic;

Modification status: None;

Genotype: Ordinary Nude Mouse.

**Experimental procedures**

9

For each experimental group, including controls, describe the procedures in enough detail to

allow others to replicate them, including:

1. What was done, how it was done and what was used.

Response: We anesthetized the mice before the following experimental operations with sodium pentobarbital, which is formulated into 1 to 3% physiological saline solution and administered by 30mg/kg BW intraperitoneal injection. Suspensions of 5 × 10^7^/0.2 mL SK-Hep1 cells were injected subcutaneously into the right hindlimbs of 5-7-week-old female BALB/c-nu/nu nude mice. When tumor volumes reached 200 mm^3^, mice were randomly assigned to control and treated groups (4 mice per group). The treated groups received 25 mg/kg PKI-587, IR, or 25 mg/kg PKI-587 combined with IR.

1. When and how often.

Response: When tumor volumes reached 200 mm^3^, mice were randomly assigned to four groups (4 mice per group). For treatment group, one group received 2Gy IR every other day; one group received 25mg/kg PKI-587 through the caudal vein every four days; one group received 25mg/kg PKI-587 in combination with 2gy IR every four days (PKI-587 was given six hours before IR exposure).

1. Where (including detail of any acclimatisation periods).

Response: All the animal experiments were carried out in SPF animal laboratory of Anhui University of Science and Technology (Anhui, People's Republic of China).

1. Why (provide rationale for procedures).

Response: All the animal experiments were carried out in strict accordance with the principles and procedures approved by the Committee on the Ethics of Animal Experiments of Anhui University of Science and Technology (Anhui, People's Republic of China).


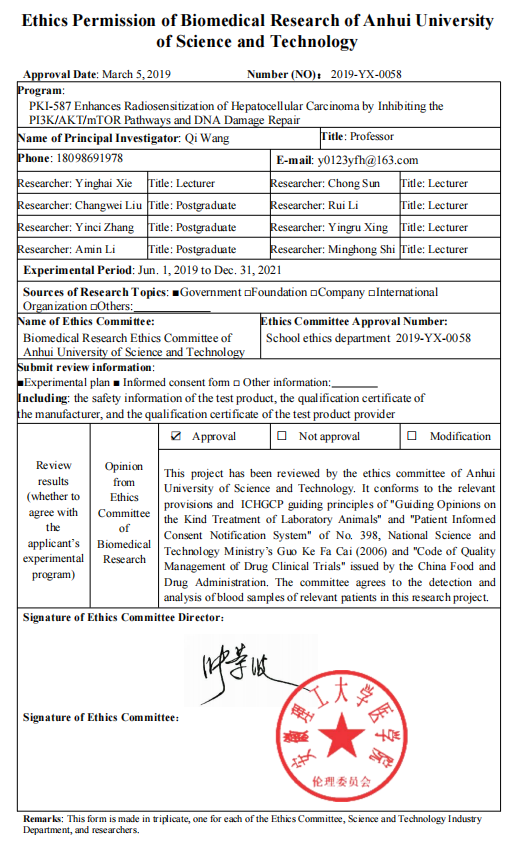


**Results**

10

For each experiment conducted, including independent replications, report:

a. Summary/descriptive statistics for each experimental group, with a measure of variability

where applicable (e.g. mean and SD, or median and range).

Response: The data were expressed as mean ± SE.

b. If applicable, the effect size with a confidence interval.

Response: The 95% confidence interval of each group of data at the end of the experiment is as follows:

Control: 2273.7500-2416.5000;

IR: 1109.7500-1807.0000;

PKI-687: 1539.7500-1921.5000;

IR+PKI-587: 300.0000-594.7500.

| **单个样本统计量** | | | | | | |
| --- | --- | --- | --- | --- | --- | --- |
|  | | Statistic | Bootstrap^a^ | | | |
|  |  |  | 偏差 | 标准 误差 | 95% 置信区间 | |
|  |  |  |  |  | 下限 | 上限 |
| Control | N | 4 |  |  |  |  |
|  | 均值 | 2340.2500 | .2392 | 34.9716 | 2273.7500 | 2416.5000 |
|  | 标准差 | 83.26814 | -15.35793 | 25.88098 | 14.50000 | 115.07987 |
|  | 均值的标准误 | 41.63407 |  |  |  |  |
| IR | N | 4 |  |  |  |  |
|  | 均值 | 1425.7500 | 2.6063 | 173.4790 | 1109.7500 | 1807.0000 |
|  | 标准差 | 411.75670 | -76.84228 | 128.86087 | 75.50000 | 497.51784 |
|  | 均值的标准误 | 205.87835 |  |  |  |  |
| PKI587 | N | 4 |  |  |  |  |
|  | 均值 | 1740.5000 | .1950 | 101.0427 | 1539.7500 | 1921.5000 |
|  | 标准差 | 238.54350 | -43.95106 | 74.43461 | 25.50000 | 289.71293 |
|  | 均值的标准误 | 119.27175 |  |  |  |  |
| Combination | N | 4 |  |  |  |  |
|  | 均值 | 428.2500 | -.9517 | 79.2784 | 300.0000 | 594.7500 |
|  | 标准差 | 182.01168 | -38.31436 | 61.09372 | 36.00000 | 221.92566 |
|  | 均值的标准误 | 91.00584 |  |  |  |  |
| a. Unless otherwise noted, bootstrap results are based on 1000 bootstrap samples | | | | | | |

**The Recommended Set**

**Abstract**

11

Provide an accurate summary of the research objectives, animal species, strain and sex,

key methods, principal findings, and study conclusions.

Response: 5-7-week-old female BALB/c-nu/nu nude mice were selected to xenograft studies. First, suspensions of 5 × 10^7^/0.2 mL SK-Hep1 cells were injected subcutaneously into the right hindlimbs of mice. When tumor volumes reached 200 mm3, mice were randomly assigned to four groups (4 mice per group). For treatment group, one group received 2Gy IR every other day; one group received 25mg/kg PKI-587 through the caudal vein every four days; one group received 25mg/kg PKI-587 in combination with 2gy IR every four days (PKI-587 was given six hours before IR exposure). The results indicated that treatment with either IR or PKI-587 slightly inhibited tumor growth, but the combination was more effective.

**Background**

12

a. Include sufficient scientific background to understand the rationale and context for the

study, and explain the experimental approach.

Response: At present, nude mice have become an indispensable experimental animal model in the field of medical biology, especially in oncology, immunology, safety evaluation of drugs and biological products and screening of effective drugs.

b. Explain how the animal species and model used address the scientific objectives and,

where appropriate, the relevance to human biology.

Response: Because of the immune deficiency in nude mice, tissue transplants from xenografts are not rejected under certain conditions. Therefore, it can be used as the recipient of human malignant tumor transplantation. Human tumor transplanted in immunodeficient animals can maintain its biological characteristics, and is suitable for the study of drug sensitivity of human tumor.

**Objectives**

13

Clearly describe the research question, research objectives and, where appropriate,

specific hypotheses being tested.

Response: To observe whether PKI-587 combined with radiotherapy can inhibit tumor growth in vivo, we conducted the xenograft studies.

**Ethical statement**

14

Provide the name of the ethical review committee or equivalent that has approved the

use of animals in this study, and any relevant licence or protocol numbers (if applicable). If

ethical approval was not sought or granted, provide a justification.

Response: Committee on the Ethics of Animal Experiments of Anhui University of Science and Technology (Anhui, People's Republic of China).

**Housing and husbandry**

15

Provide details of housing and husbandry conditions, including any environmental

enrichment.

Response: The animals live in the SPF laboratory animal room and are cared for by professional staff.

**Animal care and monitoring**

16

a. Describe any interventions or steps taken in the experimental protocols to reduce pain,

suffering and distress.

Response: In order to reduce the pain in the experimental procedure, we anesthetized the mice before experimental operations with sodium pentobarbital, which is formulated into 1 to 3% physiological saline solution and administered by 30mg/kg BW intraperitoneal injection.

b. Report any expected or unexpected adverse events.

Response: Four of the mice had failed tumor transplants.

c. Describe the humane endpoints established for the study, the signs that were

monitored and the frequency of monitoring. If the study did not have humane endpoints,

state this.

Response: The mice were euthanized with carbon dioxide. The euthanasia device, which is airtight and has good transparency, is convenient for observing whether the animal is dead during operation, is carried out in a well-ventilated environment. Before putting the mouse into the euthanasia device, a certain amount of carbon dioxide is introduced, and then the mouse is put into it, so that the mouse can enter the anesthesia state quickly and reduce fear and pain. After the animal dies, continue to infuse carbon dioxide for 2 to 3 minutes.

**Interpretation/ scientific implications**

17

a. Interpret the results, taking into account the study objectives and hypotheses, current

theory and other relevant studies in the literature.

Response: These contents have been elaborated in **Discussion**.

b. Comment on the study limitations including potential sources of bias, limitations of the

animal model, and imprecision associated with the results.

Response: These contents have been elaborated in **Discussion**.

**Generalisability/ translation**

18

Comment on whether, and how, the findings of this study are likely to generalise to other

species or experimental conditions, including any relevance to human biology (where

appropriate).

Response: The cell line we used in the study was derived from human liver cancer tissues and the animals were also mammals, so the results are applicable to species with similar biological characteristics.

**Protocol registration**

19

Provide a statement indicating whether a protocol (including the research question, key

design features, and analysis plan) was prepared before the study, and if and where this

protocol was registered.

Response: The research question, key design features, and analysis plan were prepared and registered before the study in Ethics Committee, School of Medicine, Anhui University of Science and Technology (Anhui, China).

**Data access**

20

Provide a statement describing if and where study data are available.

Response: All data have presented in the manuscript and Supporting Information files.

**Declaration of interests**

21

a. Declare any potential conflicts of interest, including financial and non-financial. If none

exist, this should be stated.

Response: All authors have no competing interests.

b. List all funding sources (including grant identifier) and the role of the funder(s) in the

design, analysis and reporting of the study.

Response: The National Natural Science Fund of China (NO.82071862, 81872017, 81572431), Anhui Provincial Science and Technology program (NO.1604a0802094, 202004j07020053), University Natural Science Research Project of Anhui Province (NO.KJ2018ZD011, KJ2018A0097, KJ2019A0093) and R&D project of Wuhu Research Institute of Anhui University of Science & Technology (NO. ALW2020YF11) funded this research. This study is part of a subset of these funded projects. All the funders provide the cost of reagents and consumables for the study.
