# Supplementary figures and images for: PKI-587 enhances radiosensitization of hepatocellular carcinoma by inhibiting the PI3K/AKT/mTOR pathways and DNA damage repair
Source: PLoS One. 2021 Oct 19;16(10):e0258817. doi: 10.1371/journal.pone.0258817 (PMC8525768; doi:10.1371/journal.pone.0258817)

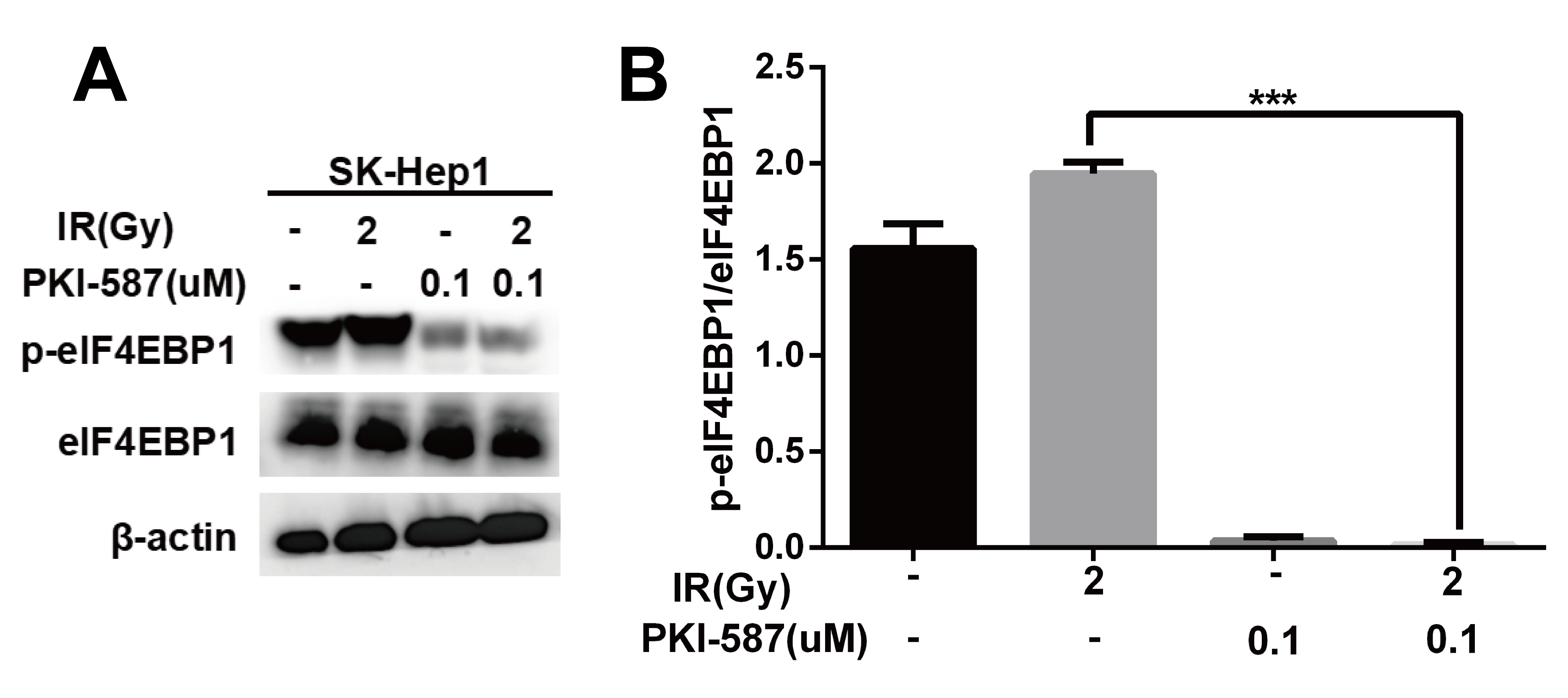

Supplement: S1 Fig — (A and B) The level of p-eIF4EBP1 and eIF4EBP1 proteins in SK-Hep1 cells after treatment with IR (2Gy) alone or combined with PKI-587 (0.1 μM) for 24 h as determined by western blot assay. The semiquantitative data were represented as p-eIF4EBP1/eIF4EBP1. The data are mean ± SD, n = 3. ***P<0.001. IR, ionizing radiation (6 MV-X ray). (TIF) [file pone.0258817.s002.tif]

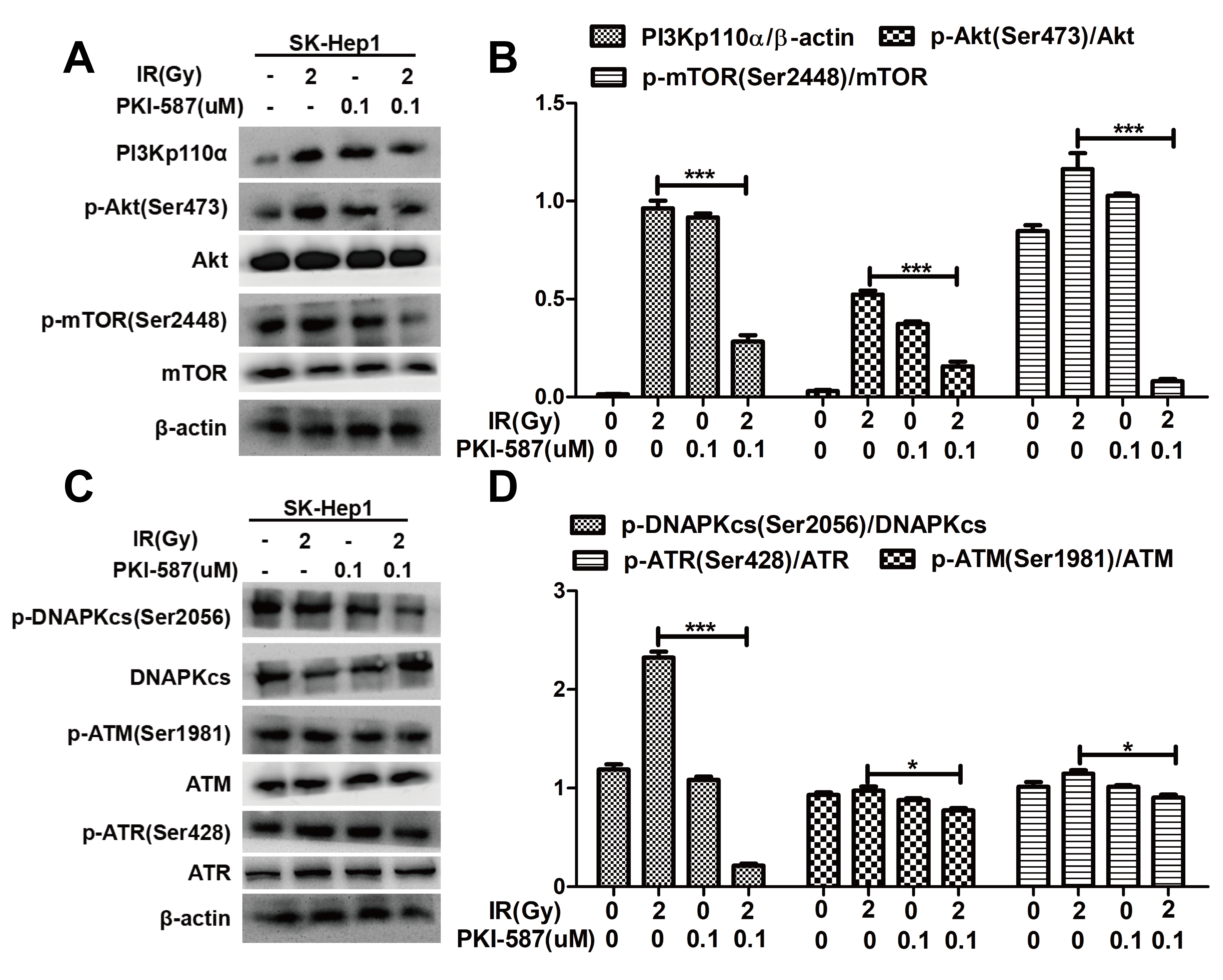

Supplement: S2 Fig — (A and B) Western blot assay and semiquantitative analysis of the levels of PI3Kp110α, p-Akt (Ser473), Akt, p-mTOR (Ser2448), and mTOR proteins involved in the PI3K/AKT/mTOR pathway in SK-Hep1 cells after radiation and PKI-587 alone or in combination for 24 h. (C and D) Western blot assay and semiquantitative analysis revealed the levels of related proteins in the DNA damage repair pathway, including p-DNAPKcs (Ser2056), DNAPKcs, p-ATR (Ser428), ATR, p-ATM (Ser1981), and ATM in SK-Hep1 cells after radiation and PKI-587 alone or in combination for 24 h. The data are mean ± SD, n = 3. *P<0.05, ***P<0.001. IR: ionizing radiation (6MV-X ray). (TIF) [file pone.0258817.s003.tif]
