# Supplementary material for: PKI-587 enhances radiosensitization of hepatocellular carcinoma by inhibiting the PI3K/AKT/mTOR pathways and DNA damage repair
Source: PLoS One. 2021 Oct 19;16(10):e0258817. doi: 10.1371/journal.pone.0258817 (PMC8525768; doi:10.1371/journal.pone.0258817)

Fig 1A

The blots are grouped from left to right: Control (0Gy IR + 0μM PKI-587), 2Gy IR + 0μM PKI-587, 0Gy IR + 0.1μM PKI-587, 2Gy IR + 0.1μM PKI-587.

PI3Kp110α 110KDa

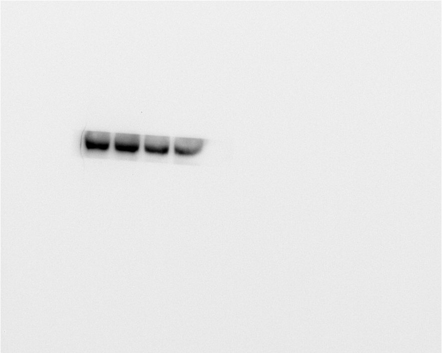

PI3Kp110r 110KDa

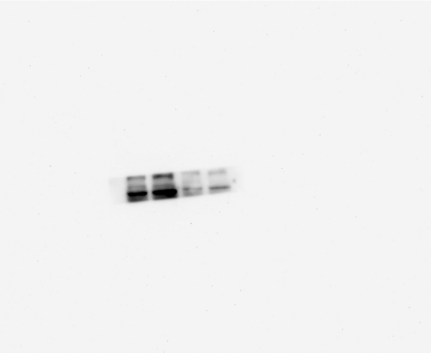

p-Akt(Ser473) 60KDa

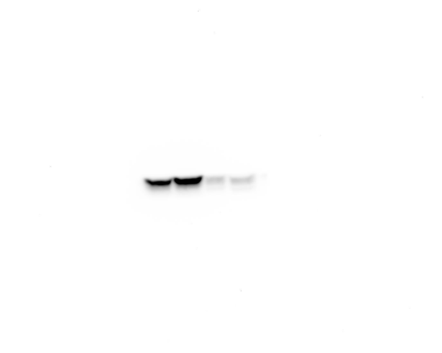

Akt 60KDa

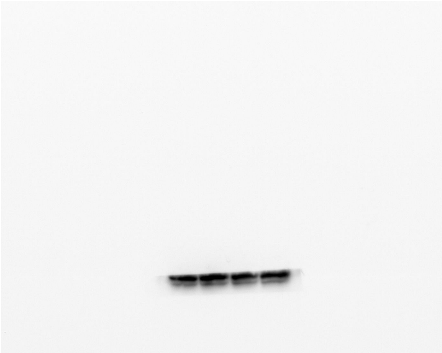

p-mTOR(Ser2448) 289KDa

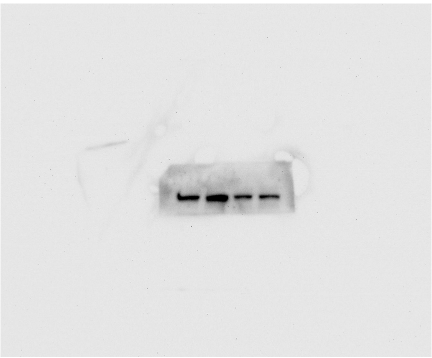

mTOR 289KDa

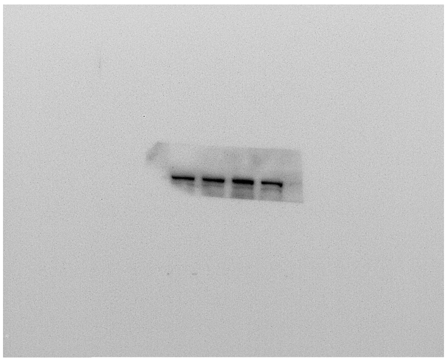

β-actin 45KDa

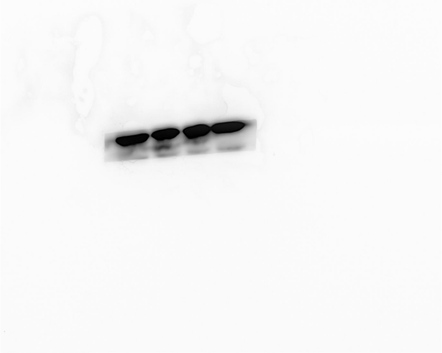

Fig 1C

The blots are grouped from left to right: Control (0Gy IR + 0μM PKI-587), 2Gy IR + 0μM PKI-587, 0Gy IR + 0.1μM PKI-587, 2Gy IR + 0.1μM PKI-587.

p-DNAPKcs(Ser2056) 469KDa

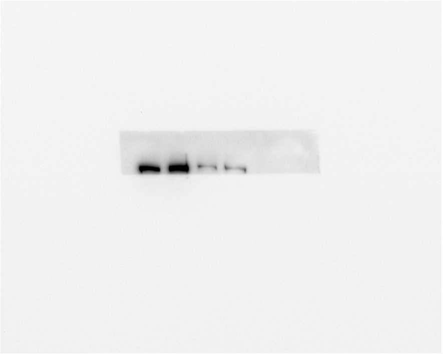

DNAPKcs 460-469KDa

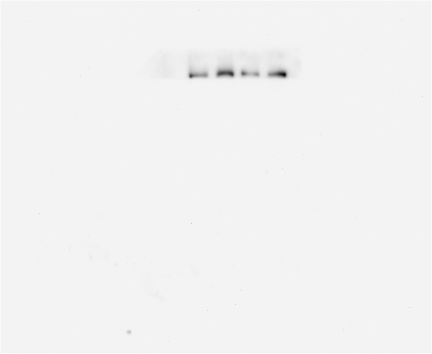

p-ATM(Ser1981) 100KDa

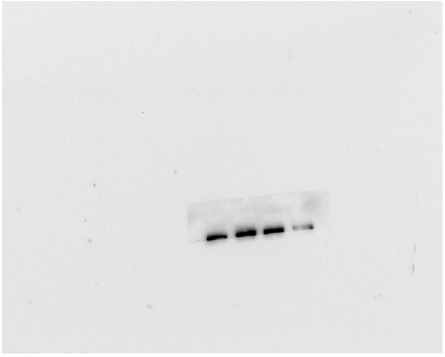

ATM 350KDa

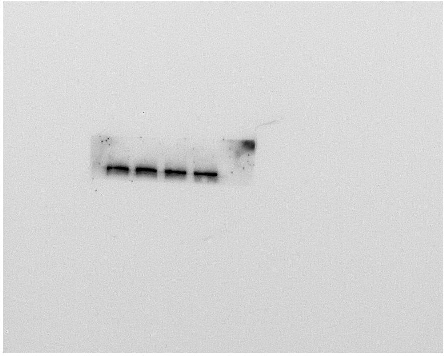

p-ATR(Ser428) 300KDa

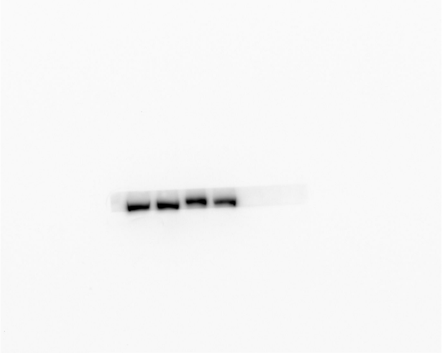

ATR 300-309KDa

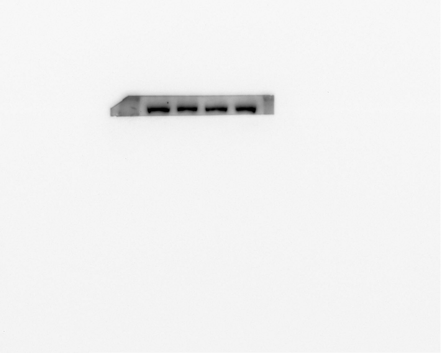

β-actin 45KDa

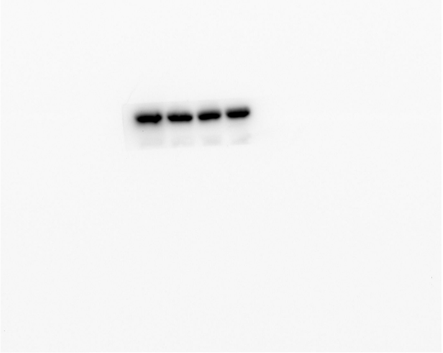

Fig 2D

The blots are grouped from left to right: Control (0Gy IR + 0μM PKI-587), 2Gy IR + 0μM PKI-587, 0Gy IR + 0.1μM PKI-587, 2Gy IR + 0.1μM PKI-587.

p-S6K1 70KDa

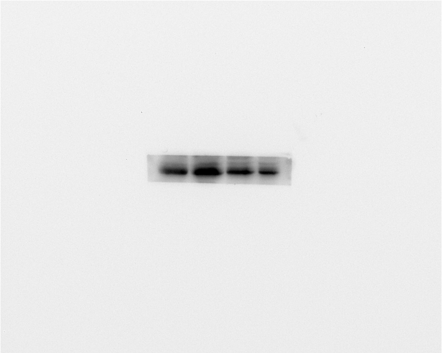

p70S6K KDa

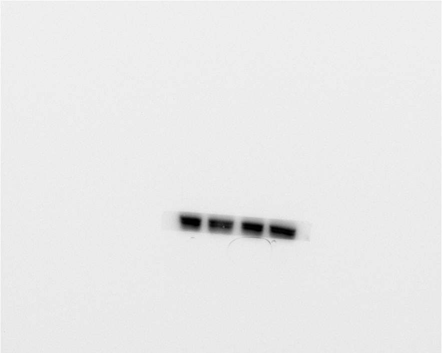

β-actin 45KDa

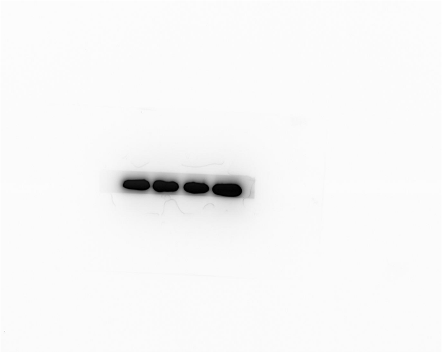

Fig 3C

The blots are grouped from left to right: 0h, 12h, 24h, 48h.

r-H2AX (2 Gy IR) 15-20KDa β-actin 45KDa

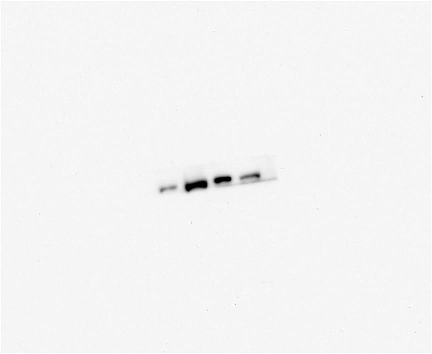

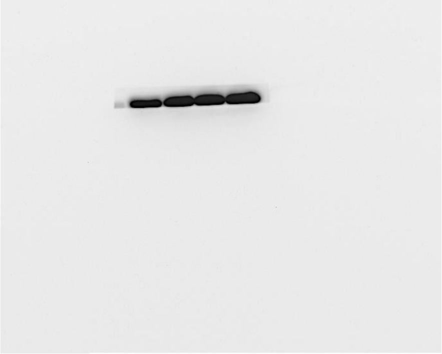

r-H2AX (2Gy IR + 0.1μM PKI-587) 15-20KDa β-actin 45KDa

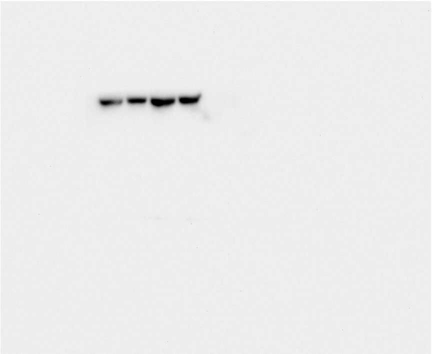

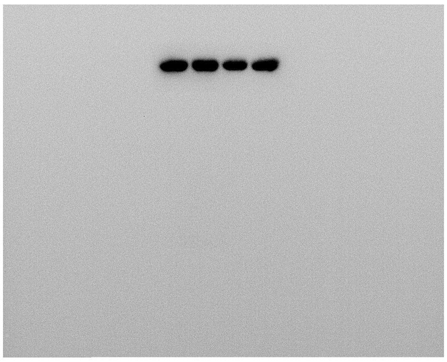

Fig 4C

The blots are grouped from left to right: Control (0Gy IR + 0μM PKI-587), 2Gy IR + 0μM PKI-587, 0Gy IR + 0.1μM PKI-587, 2Gy IR + 0.1μM PKI-587.

p-Rb 106KDa

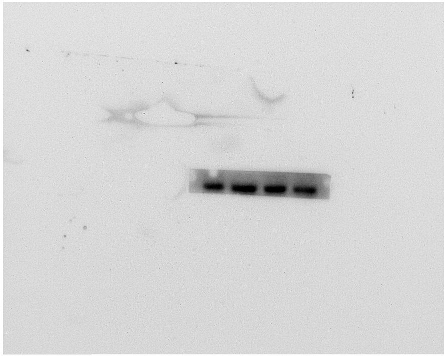

Rb 105KDa

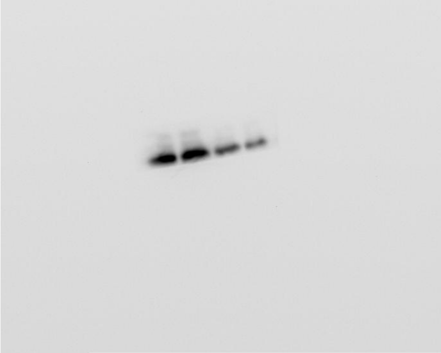

CyclinD1 33KDa

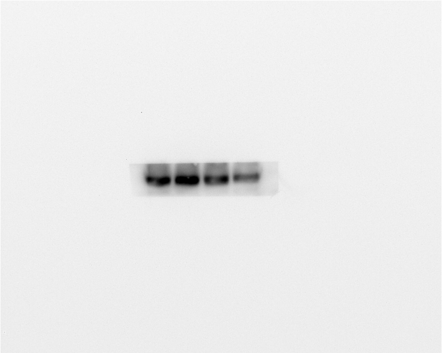

β-actin 45KDa

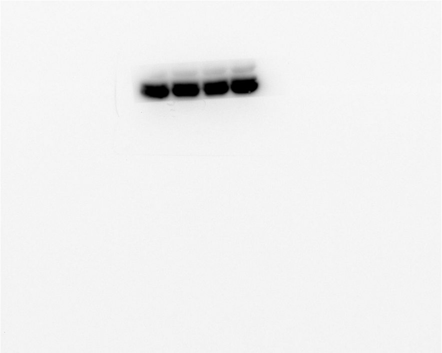

Fig 5G

The blots are grouped from left to right: Control (0Gy IR + 0μM PKI-587), 2Gy IR + 0μM PKI-587, 0Gy IR + 0.1μM PKI-587, 2Gy IR + 0.1μM PKI-587.

p-Bad 23KDa

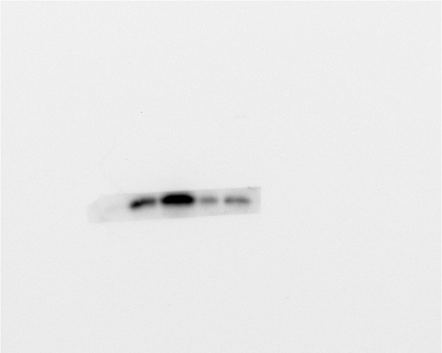

Bad 25KDa

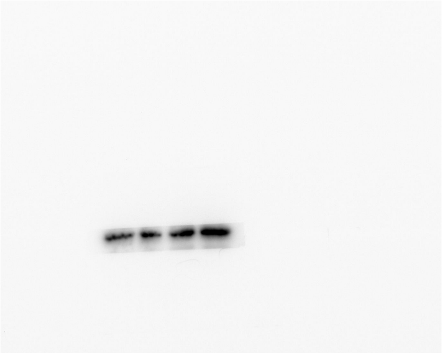

c-Caspase9 42KDa

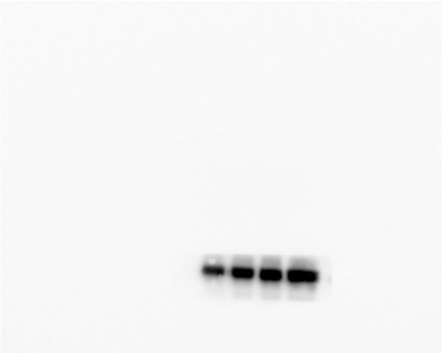

Caspase9 42KDa

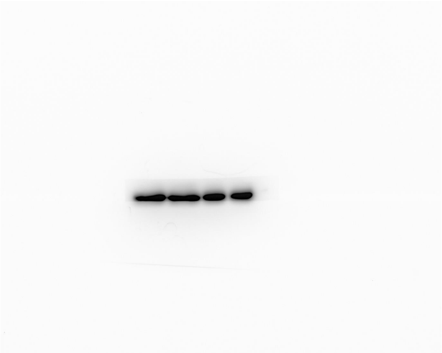

c-Caspase3 35KDa

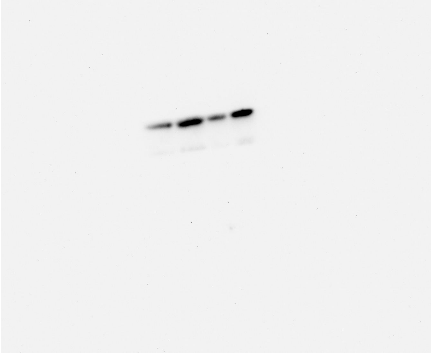

Caspase3 37KDa

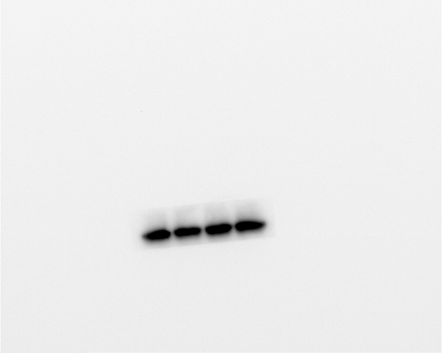

c-PARP 89KDa

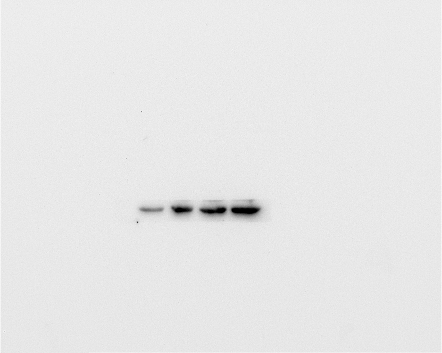

PARP 116KDa

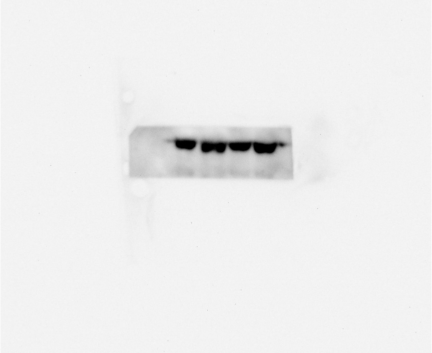

β-actin 45KDa

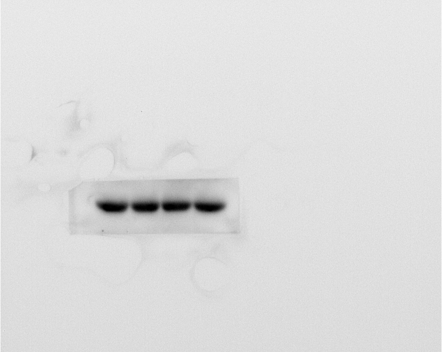

S1 Fig

The blots are grouped from left to right: Control (0Gy IR + 0μM PKI-587), 2Gy IR + 0μM PKI-587, 0Gy IR + 0.1μM PKI-587, 2Gy IR + 0.1μM PKI-587.

p-eIF4EBP1 12KDa

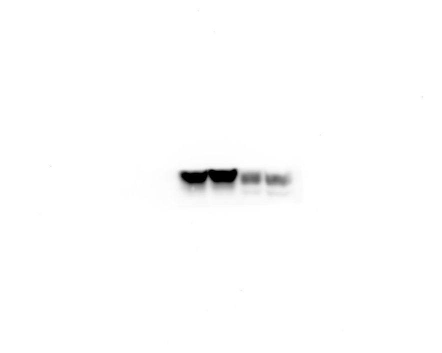

eIF4EBP1 18KDa

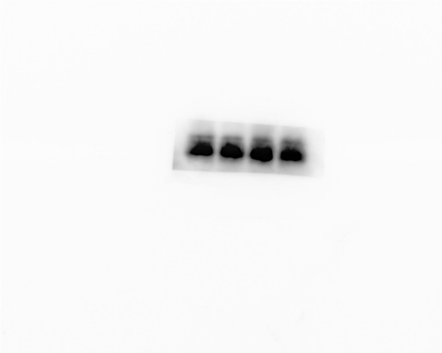

β-actin 45KDa

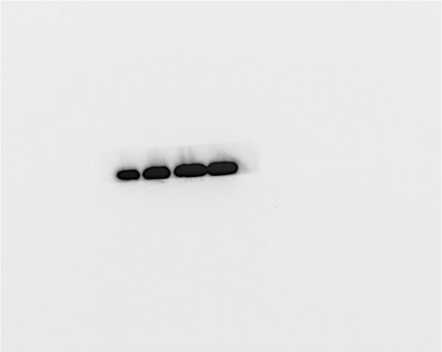

S2A Fig

The blots are grouped from left to right: Control (0Gy IR + 0mg/kg PKI-587), 2Gy IR + 0mg/kg PKI-587, 0Gy IR + 25mg/kg PKI-587, 2Gy IR + 25mg/kgPKI-587.

PI3Kp110α 110KDa

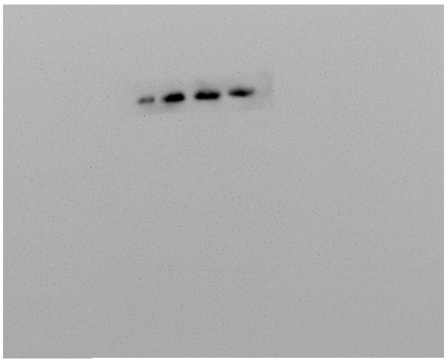

p-Akt(Ser473) 60KDa

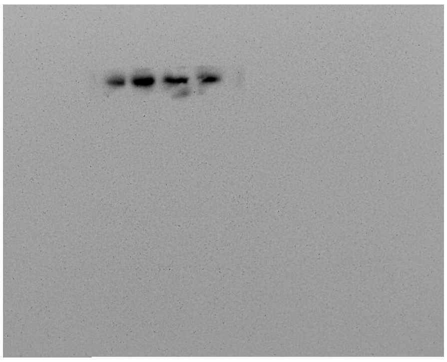

Akt 60KDa

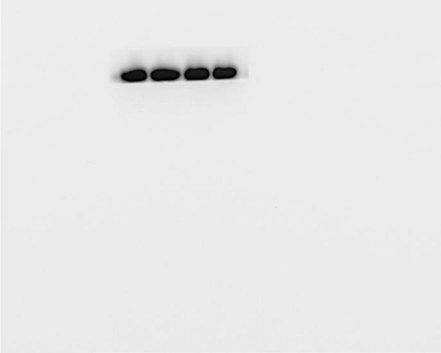

p-mTOR(Ser2448) 289KDa

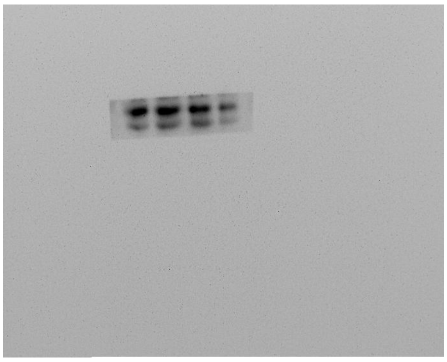

mTOR 289KDa

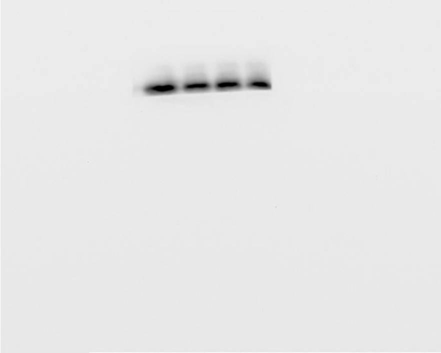

β-actin 45KDa

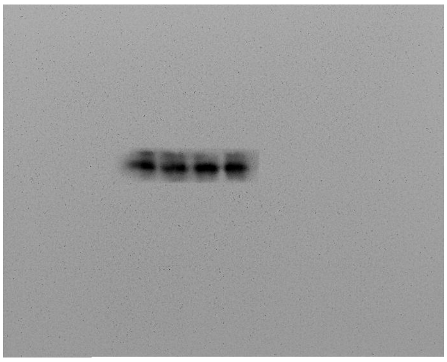

S2B Fig

The blots are grouped from left to right: Control (0Gy IR + 0mg/kg PKI-587), 2Gy IR + 0mg/kg PKI-587, 0Gy IR + 25mg/kg PKI-587, 2Gy IR + 25mg/kgPKI-587.

p-DNAPKcs(Ser2056) 469KDa

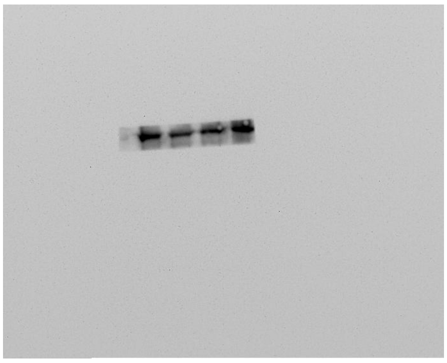

DNAPKcs 460-469KDa

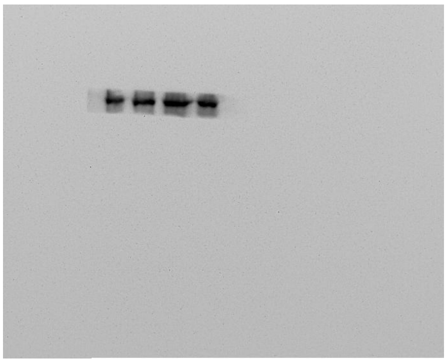

p-ATM(Ser1981) 100KDa

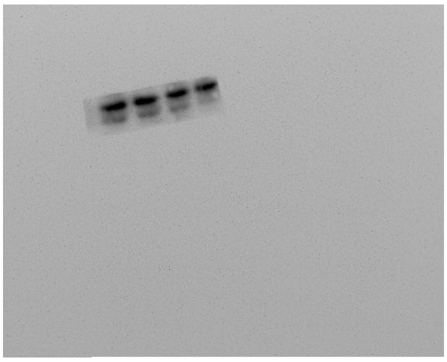

ATM 350KDa

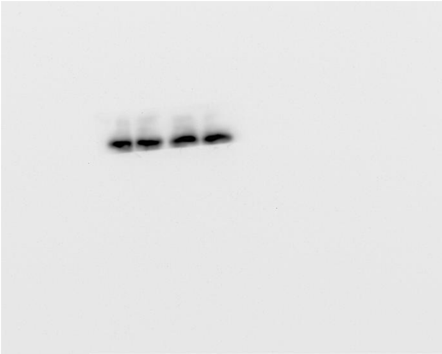

p-ATR(Ser428) 300KDa

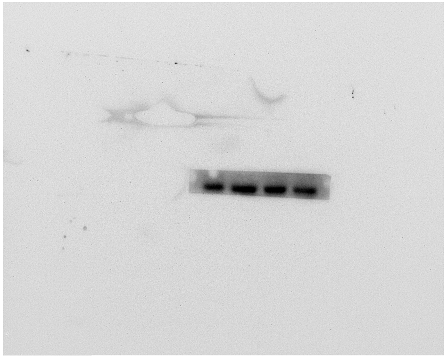

ATR 300-309KDa

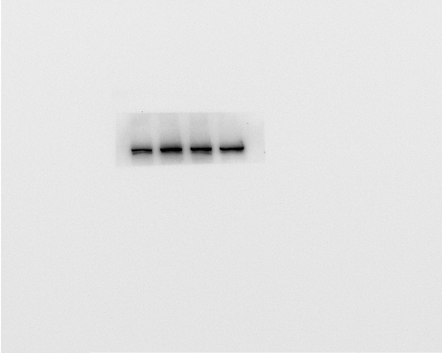

β-actin 45KDa

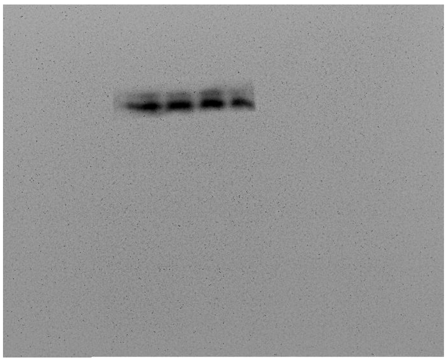

Supplement: S1 Raw images — (PDF) [file pone.0258817.s004.pdf]
