# Supplementary material for: PKI-587 enhances radiosensitization of hepatocellular carcinoma by inhibiting the PI3K/AKT/mTOR pathways and DNA damage repair
Source: PLoS One. 2021 Oct 19;16(10):e0258817. doi: 10.1371/journal.pone.0258817 (PMC8525768; doi:10.1371/journal.pone.0258817)
Supplement: S1 File — (ZIP) [file pone.0258817.s005.zip › Supporting Information files/S1_raw_images.doc]

**Fig 1A**

**The blots are grouped from left to right: Control (0Gy IR + 0μM PKI-587), 2Gy IR + 0μM PKI-587, 0Gy IR + 0.1μM PKI-587, 2Gy IR + 0.1μM PKI-587.**

PI3Kp110α	PI3Kp110r

p-Akt(Ser473)	Akt

p-mTOR(Ser2448)	mTOR

β-actin

**Fig 1C**

**The blots are grouped from left to right: Control (0Gy IR + 0μM PKI-587), 2Gy IR + 0μM PKI-587, 0Gy IR + 0.1μM PKI-587, 2Gy IR + 0.1μM PKI-587.**

p-DNAPKcs(Ser2056)	DNAPKcs

p-ATM(Ser1981)	ATM

p-ATR(Ser428)	ATR

β-actin

**Fig 2D**

**The blots are grouped from left to right: Control (0Gy IR + 0μM PKI-587), 2Gy IR + 0μM PKI-587, 0Gy IR + 0.1μM PKI-587, 2Gy IR + 0.1μM PKI-587.**

p-eIF4EBP1	eIF4EBP1

p-S6K1	p70S6K

β-actin

**Fig 3C**

**The blots are grouped from left to right: 0h, 12h, 24h, 48h.**

r-H2AX (2 Gy IR)	β-actin

r-H2AX (2Gy IR + 0.1μM PKI-587)	β-actin

**Fig 4C**

**The blots are grouped from left to right: Control (0Gy IR + 0μM PKI-587), 2Gy IR + 0μM PKI-587, 0Gy IR + 0.1μM PKI-587, 2Gy IR + 0.1μM PKI-587.**

p-Rb	Rb

CyclinD1	β-actin

**Fig 5G**

**The blots are grouped from left to right: Control (0Gy IR + 0μM PKI-587), 2Gy IR + 0μM PKI-587, 0Gy IR + 0.1μM PKI-587, 2Gy IR + 0.1μM PKI-587.**

p-Bad	Bad

c-Caspase9	Caspase9

c-Caspase3	Caspase3

c-PARP	PARP

β-actin

**Fig S1**

**The blots are grouped from left to right: Control (0Gy IR + 0μM PKI-587), 2Gy IR + 0μM PKI-587, 0Gy IR + 0.1μM PKI-587, 2Gy IR + 0.1μM PKI-587.**

p-eIF4EBP1	eIF4EBP1

β-actin

**Fig S1A**

**The blots are grouped from left to right: Control (0Gy IR + 0mg/kg PKI-587), 2Gy IR + 0mg/kg PKI-587, 0Gy IR + 25mg/kg PKI-587, 2Gy IR + 25mg/kgPKI-587.**

PI3Kp110α

p-Akt(Ser473)	Akt

p-mTOR(Ser2448)	mTOR

β-actin

**Fig S1B**

**The blots are grouped from left to right: Control (0Gy IR + 0mg/kg PKI-587), 2Gy IR + 0mg/kg PKI-587, 0Gy IR + 25mg/kg PKI-587, 2Gy IR + 25mg/kgPKI-587.**

p-DNAPKcs(Ser2056)	DNAPKcs

p-ATM(Ser1981)	ATM

p-ATR(Ser428)	ATR

β-actin
